# Study of the Three-Component Reactions of 2-Alkynylbenzaldehydes, Aniline, and Dialkyl Phosphites—The Significance of the Catalyst System

**DOI:** 10.3390/ma14206015

**Published:** 2021-10-13

**Authors:** Nóra Popovics-Tóth, Kármen Emőke Szabó, Erika Bálint

**Affiliations:** Department of Organic Chemistry and Technology, Budapest University of Technology and Economics, 1521 Budapest, Hungary; nora.toth@edu.bme.hu (N.P.-T.); szabo.karmen948@gmail.com (K.E.S.)

**Keywords:** α-aminophosphonates, dihydroisoquinolin-1-ylphosphonates, multicomponent reaction, Kabachnik–Fields reaction, T_3_P^®^

## Abstract

New, practical approaches for the synthesis of α-amino (2-alkynylphenyl)-methylphosphonates and 1,2-dihydroisoquinolin-1-ylphosphonates were developed. By the propylphosphonic anhydride (T_3_P^®^)-mediated Kabachnik–Fields reaction of 2-alkynylbenzaldehydes, aniline, and dialkyl phosphites, α-amino (2-alkynylphenyl)-methylphosphonates were obtained selectively in high yields. The method developed is a simple operation and did not require a chromatographic separation since the products could be isolated from the reaction mixture by a simple extraction. At the same time, 2,3-disubstituted-1,2-dihydroisoquinolin-1-ylphosphonates could be prepared effectively from the same kinds of starting materials (2-alkynylbenzaldehydes, aniline, and dialkyl phosphites) at 60 °C in a short reaction time by changing the catalyst for CuCl. Therefore, it was proved that the catalyst system applied played a crucial role with respect to the reaction outcome.

## 1. Introduction

Organophosphorus compounds continue to receive widespread interest due to their unique significance in organic, bio-, and medicinal chemistry, as well as in the agriculture and plastic industries [1,2,3]. One of the major classes of organophosphorus compounds is the family of organophosphates. Among them, α-aminophosphonates, as the bioisosteres and structural analogues of natural α-amino acids, have attracted a considerable focus. They were found to be effective as enzyme inhibitors, antibiotics, antiviral, antifungal, or antitumor agents, as well as pesticides [3,4,5,6,7,8]. In addition, the introduction of α-aminophosphonates into an epoxy system can improve flame retardant properties [9,10]. In recent years, the chemistry of heterocyclic derivatives of α-aminophosphonates have also received intensively growing attention [11,12,13,14]. Isoquinolines, including 1,2-dihydroisoquinolines as privileged fragments, can be considered as a common structural scaffold in several natural products that exhibit significant biological and pharmaceutical activity [15,16,17].

Multicomponent syntheses, such as the Kabachnik–Fields reaction, in which an amine, an oxo-compound, and a >P(O)H derivative react with each other, are one of the most straightforward and efficient tools for the preparation of α-aminophosphonates and their heterocyclic derivatives [18,19,20]. Applying multicomponent reactions, the target products are usually formed in a “one-pot” manner from simple starting materials with high atom economy. The ability to use various reagents makes these reactions ideal for creating new molecular libraries, and in most cases, the principles of green chemistry also prevail to save time and energy [21,22].

**Figure 1:**
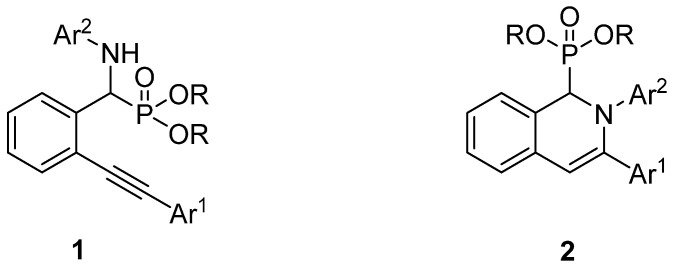
General formula of α-amino (2-alkynylphenyl)-methylphosphonates (**1**) and 1,2-dihydroisoquinolin-1-ylphosphonates (**2**).

Only a few papers have been reported on the synthesis of α-amino (2-alkynylphenyl)-methylphosphonates (**1**) and 1,2-dihydroisoquinolin-1-ylphosphonates (**2**) (Figure 1) by the three-component condensation of 2-alkynylbenzaldehydes, primary amines, and dialkyl phosphites.

Wu and co-workers performed the condensation of 2-alkynylbenzaldehyde, aromatic amines, and a small excess of diethyl phosphite in the presence of various catalytic systems [23,24,25]. It was found that in the presence of magnesium perchlorate or Lewis acid (FeCl_3_, In(OTf)_3_, Bi(OTf)_3_, Yb(OTf)_3_) in dichloroethane (DCE) at room temperature or at 60 °C for 4 h, the α-amino (2-alkynylphenyl)-methylphosphonates (**1**) were formed [23,24]; however, applying AgOTf as a catalyst in ethanol at 60 °C for 4–6 h, the cyclic phosphonates (**2**) were the main products of the reactions [23]. The latter derivatives (**2**) were also synthesized using CuI as a catalyst in DCE at 60 °C for 4 h [24] or applying a Lewis acid-type surfactant combined with a catalyst (CuSO_4_ and C_12_H_25_SO_3_Na) in water under ultrasonic conditions [25].

Recently, an enantioselective synthesis of 1,2-dihydroisoquinolin-1-ylphosphonates (**2**) was also reported [26]. In this case, the multicomponent reaction was catalyzed by a chiral silver spirocyclic phosphate in the presence of 5 Å molecular sieves in methyl tert-butyl ether at −10 °C for 3 days.

Wu and his group also described a AgOTf-catalyzed ring closure reaction of α-amino (2-alkynylphenyl)methylphosphonate (**1**), which provided the cyclic derivatives (**2**) in moderate to good yields [27].

There are two further examples of the multicomponent synthesis of 1,2-dihydroisoquinolin-1-ylphosphonates (**2**) [28,29]. In one case, 2-(2-formylphenyl)ethenone was reacted with primary amines and diethyl phosphite in the presence of CuI and 4 Å molecular sieves in DCE at 70 °C for long reaction times (12–24 h) [28]. In the other case, a multicatalytic four-component method was developed by the reaction of 2-bromobenzaldehyde, alkynes, aromatic amines, and diethyl phosphite, catalyzed by the combination of palladium and copper salts [29].

Propylphosphonic anhydride (T_3_P^®^) is a green, mild, and low toxic coupling and dehydrating agent, which delivers remarkable advantages, including broad functional group tolerance and easy work-up procedures due to the formation of water-soluble by-products, thus allowing high purity and yield for the products [30]. Several applications have been reported using this reagent, e.g., in multicomponent reactions or in the preparation of various heterocyclic compounds [31].

**Scheme 1:**
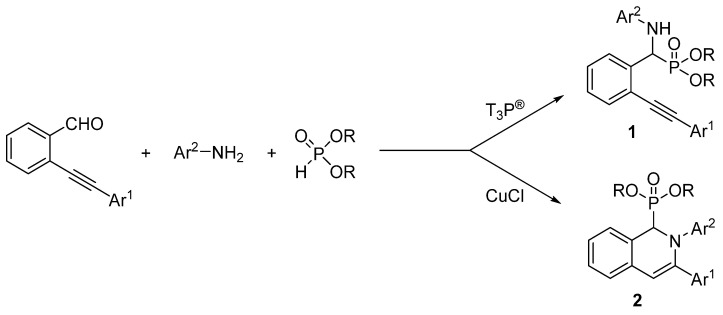
Synthesis of α-amino (2-alkynylphenyl)-methylphosphonates (**1**) and 1,2-dihydroisoquinolin-1-ylphosphonates (**2**).

In this paper, we describe simple and selective preparation methods for the synthesis of new α-amino (2-alkynylphenyl)-methylphosphonates (**1**), as well as novel 1,2-dihydroisoquinolin-1-ylphosphonates (**2**) by the three-component reaction of 2-alkynylbenzaldehydes, aromatic amines, and dialkyl phosphites using different catalytic systems (T_3_P^®^ or CuCl) (Scheme 1). There is no example in the literature of the use of T_3_P^®^ in the three-component reaction mentioned.

## 2. Materials and Methods

### 2.1. General Information

The reactions were carried out at room temperature or under conventional heating in an oil bath under N_2_ atmosphere.

High-performance liquid chromatography-mass spectrometry (HPLC-MS) measurements were performed with an Agilent 1200 liquid chromatography system coupled with a 6130 quadrupole mass spectrometer equipped with an ESI ion source (Agilent Technologies, Palo Alto, CA, USA). Analysis was performed at 40 °C on a Gemini C18 column (150 mm × 4.6 mm, 3 µm; Phenomenex, Torrance, CA, USA) with a mobile phase flow rate of 0.6 mL/min. Composition of eluent A was 0.1% (NH_4_)(HCOO) in water; eluent B was 0.1% (NH_4_)(HCOO) and 8% water in acetonitrile, 0–3 min 5% B, 3–13 min gradient, 13–20 min 100% B. The injection volume was 2 μL. The chromatographic profile was registered at 254 nm. The MSD operating parameters were as follows: positive ionization mode, scan spectra from m/z 120 to 1000, drying gas temperature 300 °C, nitrogen flow rate 12 L/min, nebulizer pressure 60 psi, capillary voltage 4000 V.

The ^31^P, ^1^H, ^13^C, NMR spectra were taken in CDCl_3_ solution on a Bruker AV-300 or DRX-500 spectrometer (Bruker AXS GmBH, Karlsruhe, Germany) operating at 121.5, 75.5, and 300 or 202.4, 125.7, and 500 MHz, respectively. Chemical shifts are downfield relative to 85% H_3_PO_4_ and TMS. Non-equivalence effect was observed in ^1^H and ^13^C{^1^H} NMR spectra. Corresponding pairs of resonances were marked with (I) and (II).

High resolution mass spectrometry measurements were performed on a Sciex TripleTOF 5600+ high resolution tandem mass spectrometer equipped with a DuoSpray ion source. Electrospray ionization was applied in positive ion detection mode. Samples were dissolved in acetonitrile and flow injected into acetonitrile/water 50:50 flow. The flow rate was 0.2 mL/min. The resolution of the mass spectrometer was 35,000.

### 2.2. General Procedure for the Synthesis of α-Amino (2-Alkynylphenyl)-Methylphosphonates (3 and 5–10)

To the mixture of 1 mmol of 2-alkynylbenzaldehydes (2-(*p*-tolylethynyl)benzaldehyde: 0.22 g, 4-fluoro-2-(*p*-tolylethynyl)benzaldehyde: 0.24 g, 2-((4-methoxyphenyl)ethynyl)-benzaldehyde: 0.24 g, 2-((4-chlorophenyl)ethynyl)benzaldehyde: 0.24 g, 2-(phenylethynyl)benzaldehyde: 0.21 g), 1 mmol (0.09 mL) of aniline and 1 mmol of dialkyl phosphites (dibutyl phosphite: 0.195 mL, dibenzyl phosphite: 0.22 mL, diethyl phosphite: 0.13 mL) was added 1.0 mmol (0.6 mL) or 0.5 mmol (0.29 mL) of T_3_P**^®^** (50% solution in EtOAc) and stirred at 25 °C or at 60 °C. After completion of the reaction (1 h), the mixture was diluted with EtOAc (15 mL) and washed with 10% NaHCO_3_ solution (15 mL). The organic phase was dried (Na_2_SO_4_), filtered, and concentrated. The following products were thus prepared:

Dibutyl ((phenylamino)(2-(p-tolylethynyl)phenyl)methyl)phosphonate (3): Yield: 96% (0.47 g), light yellow solid; Mp: 78–79 °C; ^1^H NMR (CDCl_3_) δ 0.76 (t, 3H, J_HH_ = 7.4, CH_3_^I^), 0.84 (t, 3H, J_HH_ = 7.4, CH_3_^II^), 1.13–1.22 (m, 2H, CH_2_CH_3_^I^), 1.28–1.38 (m, 4H, CH_2_CH_3_^II^, OCH_2_CH_2_^I^), 1.57–1.64 (m, 2H, OCH_2_CH_2_^II^), 2.38 (s, 3H, PhCH_3_), 3.46–3.54 (m, 1H, CH_A_, OCH_2_^I^), 3.80–3.87 (m, 1H, CH_A_, OCH_2_^II^), 4.12 (q, 2H, J_HH_ = 6.8, CH_B_, OCH_2_), 5.02 (br s, 1H, NH), 5.55 (d, 1H, ^2^J_HP_ = 24.7, CHP), 6.63–6.69 (m, 3H, ArH), 7.09 (t, 2H, J_HH_ = 7.9, ArH), 7.18 (d, 2H, J_HH_ = 7.9, ArH), 7.21–7.30 (m, 2H, ArH), 7.46 (d, 2H, J_HH_ = 8.1, ArH), 7.53 (d, 1H, J_HH_ = 7.6, ArH), 7.59 (d, 1H, J_HH_ = 7.9, ArH); ^13^C NMR (CDCl_3_) δ 13.5, 13.6, 18.5, 18.7, 21.6, 32.3 (d, ^3^*J*_CP_ = 6.0), 32.5 (d, ^3^*J*_CP_ = 5.7), 53.0 (d, ^1^*J*_CP_ = 151.3), 66.9 (d, ^2^*J*_CP_ = 7.2), 67.0 (d, ^2^*J*_CP_ = 7.4), 86.5 (d, *J*_CP_ = 2.2), 95.3, 113.6, 118.2, 120.0, 123.6 (d, ^3^*J*_CP_ = 6.9), 127.3 (d, ^3^*J*_CP_ = 4.4), 127.6 (d, *J*_CP_ = 3.1), 128.8 (d, *J*_CP_ = 3.2), 129.2, 131.4, 131.9 (d, ^3^*J*_CP_ = 2.3), 138.2 (d, *J*_CP_ = 2.2), 138.8, 146.1 (d, ^2^*J*_CP_ = 14.8); ^31^P NMR (CDCl_3_) δ 22.7; [M + H]^+^_found_ = 490.2519, [M + H]^+^_calculated_ = 490.2511.

Dibenzyl ((phenylamino)(2-(p-tolylethynyl)phenyl)methyl)phosphonate (5): Yield: 93% (0.52 g), light yellow solid; Mp: 124–125 °C; ^1^H NMR (CDCl_3_) δ 2.38 (s, 3H, PhCH_3_), 4.52 (dd, 1H, J_HH_ = 8.0, J_HH_ = 11.8, CH_A_, CH_2_O^I^), 4.84 (dd, 1H, J_HH_ = 7.4, J_HH_ = 11.8, CH_A_, CH_2_O^II^), 5.11 (d, 2H, J_HH_ = 8.2, CH_B_, CH_2_O), 5.69 (d, 1H, ^2^J_HP_ = 24.9, CHP), 6.64 (d, 2H, J_HH_ = 7.7, ArH), 6.68 (t, 1H, J_HH_ = 7.3, ArH), 7.19–7.31 (m, 10H, ArH), 7.39 (d, 2H, J_HH_ = 8.1, ArH), 7.52 (d, 1H, J_HH_ = 7.4, ArH), 7.62 (d, 1H, J_HH_ = 7.8, ArH); ^13^C NMR (CDCl_3_) δ 21.6, 53.3 (d, ^1^*J*_CP_ = 151.6), 68.48 (d, ^2^*J*_CP_ = 7.3), 68.53 (d, ^2^*J*_CP_ = 7.1), 86.5 (d, *J*_CP_ = 2.0), 95.6, 113.7, 118.4, 119.9, 123.6 (d, ^3^*J*_CP_ = 7.1), 127.4 (d, ^3^*J*_CP_ = 4.6), 127.6, 127.8 (d, *J*_CP_ = 3.1), 127.9, 128.2, 128.3, 128.4, 128.5, 128.9 (d, *J*_CP_ = 3.2), 129.23, 129.25, 131.5, 132.9 (d, ^3^*J*_CP_ = 2.2), 136.0 (d, ^3^*J*_CP_ = 6.0), 136.1 (d, ^3^*J*_CP_ = 6.0), 137.8 (d, *J*_CP_ = 1.8), 138.8, 146.0 (d, ^2^*J*_CP_ = 15.1); ^31^P NMR (CDCl_3_) δ 23.6; [M + H]^+^_found_ = 588.2205, [M + H]^+^_calculated_ = 588.2198.

Dibutyl ((4-fluoro-2-(*p*-tolylethynyl)phenyl)(phenylamino)methyl)phosphonate (6): Yield: 87% (0.44 g), light yellow solid; Mp: 72–73 °C; ^1^H NMR (CDCl_3_) δ 0.78 (t, 3H, J_HH_ = 7.4, CH_3_^I^), 0.85 (t, 3H, J_HH_ = 7.4, CH_3_^II^), 1.16–1.24 (m, 2H, CH_2_CH_3_^I^), 1.29–1.44 (m, 4H, CH_2_CH_3_^II^, OCH_2_CH_2_^I^), 1.58–1.64 (m, 2H, OCH_2_CH_2_^II^), 3.56–3.62 (m, 1H, CH_A_, OCH_2_^I^), 3.83–3.90 (m, 1H, CH_A_, OCH_2_^II^), 4.12 (q, 2H, J_HH_ = 6.8, CH_B_, OCH_2_), 4.97 (br s, 1H, NH), 5.49 (dd, 1H, ^2^J_HP_ = 24.4, J_HH_ = 4.7, CHP), 6.63 (d, 2H, J_HH_ = 7.5, ArH), 6.68 (dd, 1H, J_HH_ = 7.9, J_HH_ = 6.8, ArH), 6.99 (td, 1H, J_HH_ = 8.4, J_HH_ = 2.7, ArH), 7.10 (dd, 2H, J_HH_ = 7.2, J_HH_ = 8.6, ArH), 7.19 (d, 2H, J_HH_ = 7.9, ArH), 7.23 (dd, 1H, J_HH_ = 9.1, J_HH_ = 1.9, ArH), 7.46 (d, 2H, J_HH_ = 8.2, ArH), 7.55 (ddd, 1H, J_HH_ = 8.5, J_HH_ = 5.7, J_HH_ = 2.6, ArH); ^13^C NMR (CDCl_3_) δ 13.47, 13.55, 18.5, 18.7, 21.6, 32.3 (d, ^3^*J*_CP_ = 5.9), 32.5 (d, ^3^*J*_CP_ = 5.9), 52.5 (d, ^1^*J*_CP_ = 152.3), 66.95 (d, ^2^*J*_CP_ = 7.4), 67.02 (d, ^2^*J*_CP_ = 7.1), 85.5 (dd, *J*_CP_ = 1.8, *J*_CF_ = 3.2), 96.2, 113.6, 116.2 (dd, *J*_CP_ = 3.0, *J*_CF_ = 21.7), 118.2 (d, *J*_CP_ = 2.3), 118.4, 119.5, 125.2 (dd, ^3^*J*_CP_ = 7.0, ^3^*J*_CF_ = 9.6), 129.1 (dd, ^3^*J*_CP_ = 4.1, ^3^*J*_CF_ = 8.8), 129.28, 129.30, 131.5, 134.2 (dd, ^3^*J*_CP_ = 2.3, *J*_CF_ = 3.2), 139.3, 145.9 (d, ^2^*J*_CP_ = 14.7), 161.7 (dd, ^3^*J*_CP_ = 3.3, ^1^*J*_CF_ = 247.1); ^31^P NMR (CDCl_3_) δ 22.4; [M + H]^+^_found_ = 508.2424, [M + H]^+^_calculated_ = 508.2417.

Dibutyl ((2-((4-methoxyphenyl)ethynyl)phenyl)(phenylamino)methyl)phosphonate (7): Yield: 89% (0.45 g), light yellow oil; ^1^H NMR (CDCl_3_) δ 0.76 (t, 3H, J_HH_ = 7.4, CH_3_^I^), 0.84 (t, 3H, J_HH_ = 7.4, CH_3_^II^), 1.13–1.22 (m, 2H, CH_2_CH_3_^I^), 1.28–1.39 (m, 4H, CH_2_CH_3_^II^, OCH_2_CH_2_^I^), 1.57–1.64 (m, 2H, OCH_2_CH_2_^II^), 3.46–3.54 (m, 1H, CH_A_, OCH_2_^I^), 3.79–3.87 [3.83 (s, OCH_3_) overlapped by the multiplet of CH_A_, OCH_2_^II^ total int. 4H), 4.12 (q, 2H, J_HH_ = 6.8, CH_B_, OCH_2_), 5.03 (br s, 1H, NH), 5.54 (d, 1H, ^2^J_HP_ = 24.7, CHP), 6.62–6.68 (m, 3H, ArH), 6.89 (d, 2H, J_HH_ = 8.8, ArH), 7.09 (dd, 2H, J_HH_ = 7.4, J_HH_ = 8.4, ArH), 7.20–7.29 (m, 2H, ArH), 7.49–7.58 (m, 3H, ArH), 7.58 (d, 1H, J_HH_ = 8.0, ArH); ^13^C NMR (CDCl_3_) δ 13.5, 13.6, 18.5, 18.7, 32.3 (d, ^3^*J*_CP_ = 6.0), 32.5 (d, ^3^*J*_CP_ = 5.8), 53.0 (d, ^1^*J*_CP_ = 151.3), 55.4, 66.9 (d, ^2^*J*_CP_ = 7.5), 67.0 (d, ^2^*J*_CP_ = 7.8), 85.9 (d, *J*_CP_ = 2.3), 95.1, 113.6, 114.1, 115.2, 118.2, 123.7 (d, ^3^*J*_CP_ = 6.9), 127.3 (d, ^3^*J*_CP_ = 4.4), 127.6 (d, *J*_CP_ = 3.1), 128.6 (d, *J*_CP_ = 3.2), 129.2, 131.8 (d, ^3^*J*_CP_ = 2.6), 133.0, 138.1 (d, *J*_CP_ = 2.2), 146.2 (d, ^2^*J*_CP_ = 14.7), 159.9; ^31^P NMR (CDCl_3_) δ 22.7; [M + H]^+^_found_ = 506.2466, [M + H]^+^_calculated_ = 506.2460.

Dibutyl ((2-((4-chlorophenyl)ethynyl)phenyl)(phenylamino)methyl)phosphonate (8): Yield: 97% (0.49 g), light yellow solid; Mp: 63–64 °C; ^1^H NMR (CDCl_3_) δ 0.76 (t, 3H, J_HH_ = 7.4, CH_3_^I^), 0.85 (t, 3H, J_HH_ = 7.4, CH_3_^II^), 1.13–1.21 (m, 2H, CH_2_CH_3_^I^), 1.28–1.38 (m, 4H, CH_2_CH_3_^II^, OCH_2_CH_2_^I^), 1.57–1.64 (m, 2H, OCH_2_CH_2_^II^), 3.47–3.54 (m, 1H, CH_A_, OCH_2_^I^), 3.80–3.87 (m, 1H, CH_A_, OCH_2_^II^), 4.12 (q, 2H, J_HH_ = 6.8, CH_B_, OCH_2_), 5.01 (br s, 1H, NH), 5.49 (d, 1H, ^2^J_HP_ = 24.8, CHP), 6.62 (d, 2H, J_HH_ = 7.4, ArH), 6.67 (t, 1H, J_HH_ = 7.3, ArH), 7.09 (dd, 2H, J_HH_ = 7.2, J_HH_ = 8.6, ArH), 7.22–7.27 (m, 1H, ArH), 7.28–7.37 (m, 3H, ArH), 7.47–7.51 (m, 2H, ArH), 7.53 (d, 1H, J_HH_ = 7.6, ArH), 7.59 (d, 1H, J_HH_ = 7.9, ArH); ^13^C NMR (CDCl_3_) δ 13.5, 13.6, 18.5, 18.7, 32.3 (d, ^3^*J*_CP_ = 6.0), 32.5 (d, ^3^*J*_CP_ = 5.7), 53.2 (d, ^1^*J*_CP_ = 151.5), 66.9 (d, ^2^*J*_CP_ = 7.3), 67.0 (d, ^2^*J*_CP_ = 7.4), 88.1 (d, *J*_CP_ = 2.1), 93.9, 113.6, 118.3, 121.6, 123.0 (d, ^3^*J*_CP_ = 6.8), 127.4 (d, ^3^*J*_CP_ = 4.3), 127.7 (d, *J*_CP_ = 3.0), 128.8, 129.2 (d, *J*_CP_ = 3.1), 129.3, 132.0 (d, ^3^*J*_CP_ = 2.2), 132.7, 134.7, 138.5 (d, *J*_CP_ = 2.2), 146.1 (d, ^2^*J*_CP_ = 14.6); ^31^P NMR (CDCl_3_) δ 22.6; [M + H]^+^_found_ = 510.1974, [M + H]^+^_calculated_ = 510.1965.

Dibutyl ((phenylamino)(2-(phenylethynyl)phenyl)methyl)phosphonate (9): Yield: 98% (0.47 g), light yellow solid; Mp: 97–98 °C; ^1^H NMR (CDCl_3_) δ 0.76 (t, 3H, J_HH_ = 7.4, CH_3_^I^), 0.84 (t, 3H, J_HH_ = 7.4, CH_3_^II^), 1.13–1.22 (m, 2H, CH_2_CH_3_^I^), 1.28–1.40 (m, 4H, CH_2_CH_3_^II^, OCH_2_CH_2_^I^), 1.57–1.64 (m, 2H, OCH_2_CH_2_^II^), 3.46–3.55 (m, 1H, CH_A_, OCH_2_^I^), 3.79–3.87 (m, 1H, CH_A_, OCH_2_^II^), 4.12 (q, 2H, J_HH_ = 6.8, CH_B_, OCH_2_), 5.02 (br s, 1H, NH), 5.55 (dd, 1H, ^2^J_HP_ = 24.7, J_HH_ = 6.8, CHP), 6.63–6.69 (m, 3H, ArH), 7.09 (dd, 2H, J_HH_ = 7.2, J_HH_ = 8.7, ArH), 7.24–7.27 (m, 1H, ArH), 7.30 (t, 1H, J_HH_ = 7.6, ArH), 7.35–7.39 (m, 3H, ArH), 7.52–7.61 (m, 4H, ArH); ^13^C NMR (CDCl_3_) δ 13.48, 13.55, 18.5, 18.7, 21.6, 32.3 (d, ^3^*J*_CP_ = 6.0), 32.5 (d, ^3^*J*_CP_ = 5.8), 53.0 (d, ^1^*J*_CP_ = 151.2), 66.9 (d, ^2^*J*_CP_ = 7.2), 67.0 (d, ^2^*J*_CP_ = 7.4), 87.2 (d, *J*_CP_ = 2.3), 95.0, 113.6, 118.2, 123.1, 123.3 (d, ^3^*J*_CP_ = 6.9), 127.4 (d, ^3^*J*_CP_ = 4.3), 127.7 (d, *J*_CP_ = 3.2), 128.5, 128.6, 129.0 (d, *J*_CP_ = 3.1), 129.3, 131.5, 132.0 (d, ^3^*J*_CP_ = 2.2), 138.4 (d, *J*_CP_ = 2.2), 146.1 (d, ^2^*J*_CP_ = 15.0); ^31^P NMR (CDCl_3_) δ 22.6; [M + H]^+^_found_ = 476.2366, [M + H]^+^_calculated_ = 476.2355.

Diethyl ((phenylamino)(2-(phenylethynyl)phenyl)methyl)phosphonate (10): Yield: 95% (0.40 g), light yellow solid; Mp: 135–136 °C; ^1^H NMR (CDCl_3_) δ 1.05 (t, 3H, J_HH_ = 7.1, CH_3_^I^), 1.30 (t, 3H, J_HH_ = 7.1, CH_3_^II^), 3.56–3.66 (m, 1H, CH_A_, OCH_2_^I^), 3.85–3.94 (m, 1H, CH_A_, OCH_2_^II^), 4.21 (q, 2H, J_HH_ = 7.3, CH_B_, OCH_2_), 5.01 (br s, 1H, NH), 5.55 (d, 1H, ^2^J_HP_ = 24.8, CHP), 6.62–6.70 (m, 3H, ArH), 7.10 (t, 2H, J_HH_ = 7.8, ArH), 7.22–7.28 (m, 1H, ArH), 7.30 (t, 1H, J_HH_ = 7.5, ArH), 7.34–7.42 (m, 3H, ArH), 7.53–7.62 (m, 4H, ArH);^13^C NMR (CDCl_3_) δ 16.1 (d, ^3^*J*_CP_ = 5.8), 16.5 (d, ^3^*J*_CP_ = 5.8), 53.2 (d, ^1^*J*_CP_ = 151.3), 63.2 (d, ^2^*J*_CP_ = 6.9), 63.5 (d, ^2^*J*_CP_ = 7.2), 87.1 (d, *J*_CP_ = 2.1), 95.1, 113.6, 118.3, 123.1, 123.4 (d, ^3^*J*_CP_ = 6.9), 127.4 (d, ^3^*J*_CP_ = 4.4), 127.7 (d, *J*_CP_ = 3.1), 128.5, 128.6, 129.0 (d, *J*_CP_ = 3.1), 129.3, 131.5, 132.1 (d, ^3^*J*_CP_ = 2.3), 138.3 (d, *J*_CP_ = 2.4), 146.1 (d, ^2^*J*_CP_ = 14.8); ^31^P NMR (CDCl_3_) δ 22.7; [M + H]^+^_found_ = 420.1736, [M + H]^+^_calculated_ = 420.1729.

### 2.3. General Procedure for the Synthesis of 1,2-Dihydroisoquinolin-1-Ylphosphonates (4 and 11–18)

To the mixture of 2-alkynylbenzaldehydes [(2-(*p*-tolylethynyl)benzaldehyde: 1 mmol (0.22 g) or 1.2 mmol (0.26 g), 4-fluoro-2-(*p*-tolylethynyl)benzaldehyde: 1.2 mmol (0.29 g), 2-((4-methoxyphenyl)ethynyl)benzaldehyde: 1.2 mmol (0.29 g), 2-((4-chlorophenyl)ethynyl)benzaldehyde: 1.2 mmol (0.29 g), 2-(phenylethynyl)benzaldehyde: 1.2 mmol (0.25 g)], amine [(aniline: 1 mmol (0.09 mL), 1.2 mmol (0.11 mL), *p*-anisidine: 1.2 mmol (0.15 g), 4-chloroaniline: 1.2 mmol (0.15 g)] and 1 mmol of dialkyl phosphites (dibutyl phosphite: 0.195 mL, dimethyl phosphite: 0.09 mL, diethyl phosphite: 0.13 mL) was added copper catalyst [0.05 mmol (5 mg) or 0.10 mmol (10 mg) of CuCl, 0.10 mmol (25 mg) CuSO_4_·5H_2_O, 0.10 mmol (12 mg) of CuBr or 0.10 mmol (19 mg) of CuI)] in 1 mL of acetonitrile under N_2_ atmosphere. The mixture was stirred at 60 °C. The volatile components were removed in vacuum, and the residue was analyzed by ^31^P NMR spectroscopy and by HPLC-MS. The 1,2-dihydroisoquinolin-1-ylphosphonates were obtained after column chromatography using silica gel as the absorbent and dichloromethane/methanol (99:1) as the eluent. The following products were thus prepared:

Dibutyl (2-phenyl-3-(*p*-tolyl)-1,2-dihydroisoquinolin-1-yl)phosphonate (4): Yield: 79% (0.39 g), yellow oil; ^1^H NMR (CDCl_3_) δ 0.83 (t, 3H, J_HH_ = 7.5, CH_3_^I^), 0.84 (t, 3H, J_HH_ = 7.3, CH_3_^II^), 1.23–1.34 (m, 4H, CH_2_CH_3_), 1.47–1.59 (m, 4H, OCH_2_CH_2_), 2.27 (s, 3H, PhCH_3_), 3.79–3.91 (m, 2H, OCH_2_^I^), 3.91–3.98 (m, 1H, CH_A_, OCH_2_^II^), 3.98–4.06 (m, 1H, CH_B_, OCH_2_^II^), 5.44 (d, 1H, ^2^J_HP_ = 18.8, CHP), 6.46 (s, 1H, ArH), 6.82–6.88 (m, 1H, ArH), 7.03 (d, 2H, J_HH_ = 7.6, ArH), 7.05–7.20 (m, 7H, ArH), 7.20–7.27 (m, 1H, ArH), 7.46 (d, 2H, J_HH_ = 7.7, ArH); ^13^C NMR (CDCl_3_) δ 13.50, 13.53, 18.60, 18.62, 21.2, 32.56 (d, ^3^*J*_CP_ = 5.4), 32.59 (d, ^3^*J*_CP_ = 5.7), 64.2 (d, ^1^*J*_CP_ = 163.6), 66.1 (d, ^2^*J*_CP_ = 7.4), 66.4 (d, ^2^*J*_CP_ = 7.6), 111.6, 122.2, 122.63, 122.65, 124.2 (d, *J*_CP_ = 2.7), 125.7 (d, *J*_CP_ = 3.2), 126.4 (d, *J*_CP_ = 2.0), 127.2 (d, ^2^*J*_CP_ = 5.9), 127.5, 128.2 (d, *J*_CP_ = 3.1), 128.4, 129.0, 133.2 (d, ^3^*J*_CP_ = 3.3), 134.5, 137.7, 142.0 (d, ^3^*J*_CP_ = 1.8), 147.8 (d, ^3^*J*_CP_ = 7.2); ^31^P NMR (CDCl_3_) δ 20.8; [M + H]^+^_found_ = 490.2517, [M + H]^+^_calculated_ = 490.2511.

Dimethyl (2-phenyl-3-(*p*-tolyl)-1,2-dihydroisoquinolin-1-yl)phosphonate (11): Yield: 86% (0.35 g), yellow oil; ^1^H NMR (CDCl_3_) δ 2.27 (s, 3H, PhCH_3_), 3.61 (d, 3H, J_HH_ = 10.5, OCH_3_^I^), 3.68 (d, 3H, J_HH_ = 10.6, OCH_3_^II^), 5.47 (d, 1H, ^2^J_HP_ = 18.7, CHP), 6.50 (s, 1H, ArH), 6.84–6.89 (m, 1H, ArH), 7.03–7.21 (m, 9H, ArH), 7.22–7.27 (m, 1H, ArH), 7.46 (d, 2H, J_HH_ = 8.2, ArH); ^13^C NMR (CDCl_3_) δ 21.2, 53.27 (d, ^2^*J*_CP_ = 9.5), 53.32 (d, ^2^*J*_CP_ = 8.9), 64.0 (d, ^1^*J*_CP_ = 163.3), 111.4, 122.5, 122.80, 122.82, 124.3 (d, *J*_CP_ = 2.7), 125.4 (d, *J*_CP_ = 3.2), 126.6 (d, *J*_CP_ = 2.1), 127.2 (d, ^2^*J*_CP_ = 6.0), 127.6, 128.4 (d, *J*_CP_ = 3.1), 128.6, 129.1, 133.2 (d, ^3^*J*_CP_ = 3.2), 134.4, 138.0, 142.2 (d, ^3^*J*_CP_ = 1.8), 147.7 (d, ^3^*J*_CP_ = 7.1); ^31^P NMR (CDCl_3_) δ 23.2; [M + H]^+^_found_ = 406.1580, [M + H]^+^_calculated_ = 406.1572.

Diethyl (2-phenyl-3-(*p*-tolyl)-1,2-dihydroisoquinolin-1-yl)phosphonate (12): Yield: 83% (0.36 g), yellow oil; ^1^H NMR (CDCl_3_) δ 1.19 (t, 3H, J_HH_ = 7.0, CH_3_^I^), 1.23 (t, 3H, J_HH_ = 7.2, CH_3_^II^), 2.27 (s, 3H, PhCH_3_), 3.86–3.98 (m, 2H, OCH_2_^I^), 3.98–4.04 (m, 1H, CH_A_, OCH_2_^II^), 4.06–4.13 (m, 1H, CH_B_, OCH_2_^II^), 5.43 (d, 1H, ^2^J_HP_ = 18.8, CHP), 6.47 (s, 1H, ArH), 6.82–6.88 (m, 1H, ArH), 7.04 (d, 2H, J_HH_ = 7.8, ArH), 7.06–7.20 (m, 7H, ArH), 7.21–7.27 (m, 1H, ArH), 7.47 (d, 2H, J_HH_ = 7.8, ArH); ^13^C NMR (CDCl_3_) δ 16.4 (d, ^3^*J*_CP_ = 5.4), 16.5 (d, ^3^*J*_CP_ = 5.6), 21.2, 62.5 (d, ^2^*J*_CP_ = 7.1), 62.7 (d, ^2^*J*_CP_ = 7.5), 64.2 (d, ^1^*J*_CP_ = 163.2), 111.5, 122.2, 122.70, 122.72, 124.2 (d, *J*_CP_ = 2.7), 125.6 (d, *J*_CP_ = 3.2), 126.4 (d, *J*_CP_ = 2.0), 127.2 (d, ^2^*J*_CP_ = 5.9), 127.5, 128.2 (d, *J*_CP_ = 3.1), 128.5, 129.0, 133.2 (d, ^3^*J*_CP_ = 3.1), 134.5, 137.8, 142.1 (d, ^3^*J*_CP_ = 1.1), 147.8 (d, ^3^*J*_CP_ = 7.2); ^31^P NMR (CDCl_3_) δ 20.9; [M + H]^+^_found_ = 434.1894, [M + H]^+^_calculated_ = 434.1885.

Dibutyl (6-fluoro-2-phenyl-3-(*p*-tolyl)-1,2-dihydroisoquinolin-1-yl)phosphonate (13): Yield: 81% (0.41 g), yellow oil; ^1^H NMR (CDCl_3_) δ 0.83 (t, 3H, J_HH_ = 7.4, CH_3_^I^), 0.85 (t, 3H, J_HH_ = 7.4, CH_3_^II^), 1.24–1.34 (m, 4H, CH_2_CH_3_), 1.49–1.59 (m, 4H, OCH_2_CH_2_), 2.27 (s, 3H, PhCH_3_), 3.84–4.05 (m, 4H, OCH_2_), 5.39 (d, 1H, ^2^J_HP_ = 18.4, CHP), 6.38 (s, 1H, ArH), 6.79–6.89 (m, 3H, ArH), 7.01–7.12 (m, 7H, ArH), 7.49 (d, 2H, J_HH_ = 8.3, ArH); ^13^C NMR (CDCl_3_) δ 13.5, 13.6, 18.66, 18.68, 21.2, 32.6 (d, ^3^*J*_CP_ = 5.6), 32.7 (d, ^3^*J*_CP_ = 5.7), 63.8 (d, ^1^*J*_CP_ = 164.9), 66.3 (d, ^2^*J*_CP_ = 7.3), 66.4 (d, ^2^*J*_CP_ = 7.6), 110.5, (dd, *J*_CP_ = 2.5, ^2^*J*_CF_ = 22.6), 110.6 (d, *J*_CP_ = 2.4), 113.0 (dd, *J*_CP_ = 1.8, ^2^*J*_CF_ = 22.4), 121.2 (t, *J*_CP_ = 3.0), 122.6, 122.91, 122.93, 127.7, 128.57, 128.60 (dd, ^2^*J*_CP_ = 6.0, *J*_CF_ = 8.7), 129.1, 134.1, 135.2 (d, ^3^*J*_CP_ = 3.1, ^3^*J*_CF_ = 8.7), 138.2, 143.3 (d, ^3^*J*_CP_ = 1.7), 147.7 (d, ^3^*J*_CP_ = 6.9), 162.9 (dd, *J*_CP_ = 3.1, ^1^*J*_CF_ = 244.9); ^31^P NMR (CDCl_3_) δ 20.5; [M + H]^+^_found_ = 508.2424, [M + H]^+^_calculated_ = 508.2417.

Dibutyl (3-(4-methoxyphenyl)-2-phenyl-1,2-dihydroisoquinolin-1-yl)phosphonate (14): Yield: 80% (0.40 g), yellow oil; ^1^H NMR (CDCl_3_) δ 0.82 (t, 3H, J_HH_ = 7.4, CH_3_^I^), 0.84 (t, 3H, J_HH_ = 7.4, CH_3_^II^), 1.24–1.33 (m, 4H, CH_2_CH_3_), 1.48–1.58 (m, 4H, OCH_2_CH_2_), 3.74 (s, 3H, PhOCH_3_), 3.81–4.02 (m, 4H, OCH_2_), 5.42 (d, 1H, ^2^J_HP_ = 18.9, CHP), 6.41 (s, 1H, ArH), 6.75 (d, 2H, J_HH_ = 8.7, ArH), 6.83–6.87 (m, 1H, ArH), 7.05–7.18 (m, 7H, ArH), 7.20–7.25 (m, 1H, ArH), 7.49 (d, 2H, J_HH_ = 8.8, ArH); ^13^C NMR (CDCl_3_) δ 13.56, 13.59, 18.60, 18.67, 32.61 (d, ^3^*J*_CP_ = 5.6), 32.64 (d, ^3^*J*_CP_ = 5.6), 55.2, 64.2 (d, ^1^*J*_CP_ = 163.5), 66.2 (d, ^2^*J*_CP_ = 7.3), 66.4 (d, ^2^*J*_CP_ = 7.7), 111.0, 113.7, 122.2, 122.75, 122.77, 124.1 (d, *J*_CP_ = 2.7), 125.7 (d, *J*_CP_ = 3.2), 126.3 (d, *J*_CP_ = 2.2), 127.2 (d, ^2^*J*_CP_ = 5.9), 128.2 (d, *J*_CP_ = 3.2), 128.5, 128.9, 129.4, 133.3 (d, ^3^*J*_CP_ = 3.1), 141.8 (d, ^3^*J*_CP_ = 1.8), 147.9 (d, ^3^*J*_CP_ = 7.2), 159.4; ^31^P NMR (CDCl_3_) δ 20.9; [M + H]^+^_found_ = 506.2469, [M + H]^+^_calculated_ = 506.2460.

Dibutyl (3-(4-chlorophenyl)-2-phenyl-1,2-dihydroisoquinolin-1-yl)phosphonate (15): Yield: 82% (0.42 g), yellow oil; ^1^H NMR (CDCl_3_) δ 0.82 (t, 3H, J_HH_ = 7.3, CH_3_^I^), 0.84 (t, 3H, J_HH_ = 7.5, CH_3_^II^), 1.24–1.33 (m, 4H, CH_2_CH_3_), 1.47–1.59 (m, 4H, OCH_2_CH_2_), 3.81–4.02 (m, 2H, OCH_2_), 5.41 (d, 1H, ^2^J_HP_ = 18.8, CHP), 6.48 (s, 1H, ArH), 6.87 (t, 1H, J_HH_ = 7.1, ArH), 7.04 (d, 2H, J_HH_ = 8.1, ArH), 7.08–7.13 (m, 3H, ArH), 7.14–7.22 (m, 4H, ArH), 7.23–7.28 (m, 1H, ArH), 7.50 (d, 2H, J_HH_ = 8.6, ArH); ^13^C NMR (CDCl_3_) δ 13.5, 13.6, 18.65, 18.66, 32.6 (d, ^3^*J*_CP_ = 5.4), 32.7 (d, ^3^*J*_CP_ = 5.7), 64.1 (d, ^1^*J*_CP_ = 163.8), 66.2 (d, ^2^*J*_CP_ = 7.4), 66.4 (d, ^2^*J*_CP_ = 7.7), 112.8, 122.58, 122.62, 122.64, 124.5 (d, *J*_CP_ = 2.7), 125.8 (d, *J*_CP_ = 3.2), 126.9 (d, *J*_CP_ = 2.0), 127.3 (d, ^2^*J*_CP_ = 5.9), 128.3 (d, *J*_CP_ = 3.2), 128.6, 128.7, 128.8, 132.8 (d, ^3^*J*_CP_ = 3.3), 133.6, 136.0, 140.9 (d, ^3^*J*_CP_ = 1.8), 147.5 (d, ^3^*J*_CP_ = 7.1); ^31^P NMR (CDCl_3_) δ 20.7; [M + H]^+^_found_ = 510.1974, [M + H]^+^_calculated_ = 510.1965.

Dibutyl (2,3-diphenyl-1,2-dihydroisoquinolin-1-yl)phosphonate (16): Yield: 80% (0.38 g), yellow oil; ^1^H NMR (CDCl_3_) δ 0.86 (t, 3H, J_HH_ = 7.1, CH_3_^I^), 0.87 (t, 3H, J_HH_ = 7.2, CH_3_^II^), 1.27–1.36 (m, 4H, CH_2_CH_3_), 1.50–1.62 (m, 4H, OCH_2_CH_2_), 3.84–4.07 (m, 4H, OCH_2_), 5.48 (d, 1H, ^2^J_HP_ = 18.8, CHP), 6.53 (s, 1H, ArH), 6.85–6.91 (m, 1H, ArH), 7.08–7.30 (m, 11H, ArH), 7.61 (d, 2H, J_HH_ = 7.7, ArH); ^13^C NMR (CDCl_3_) δ 13.5, 13.6, 18.65, 18.66, 32.61 (d, ^3^*J*_CP_ = 5.7), 32.63 (d, ^3^*J*_CP_ = 5.6), 64.2 (d, ^1^*J*_CP_ = 163.6), 66.2 (d, ^2^*J*_CP_ = 7.2), 66.4 (d, ^2^*J*_CP_ = 7.7), 112.4, 122.3, 122.63, 122.65, 124.3 (d, *J*_CP_ = 2.8), 125.8 (d, *J*_CP_ = 3.5), 126.6 (d, *J*_CP_ = 2.6), 127.3 (d, ^2^*J*_CP_ = 6.0), 127.6, 127.9.5, 128.26, 128.27 (d, *J*_CP_ = 2.3), 128.5, 133.1 (d, ^3^*J*_CP_ = 3.6), 137.4, 142.0 (d, ^3^*J*_CP_ = 1.9), 147.8 (d, ^3^*J*_CP_ = 7.7); ^31^P NMR (CDCl_3_) δ 20.8; [M + H]^+^_found_ = 476.2362, [M + H]^+^_calculated_ = 476.2355.

## 3. Results and Discussion

First, the model reaction of 2-(*p*-tolylethynyl)benzaldehyde (**A**), aniline (**B**), and dibutyl phosphite (**C**) was studied (Table 1). Performing the three-component condensation without any catalyst in acetonitrile at 60 °C for 4 h, the conversion was only 52%, and the dibutyl ((phenylamino)(2-(*p*-tolylethynyl)phenyl)methyl)phosphonate (**3**) was the main product; however, 2% of dibutyl (2-phenyl-3-(*p*-tolyl)-1,2-dihydroisoquinolin-1-yl)phosphonate (**4**) was also formed (Table 1, Entry 1). Repeating the reaction in the presence of half equivalent of propylphosphonic anhydride (T_3_P^®^) as the condensing agent, at room temperature for 1 h, it was found that a conversion of 70% could be already reached, and product **3** was formed selectively (Table 1, Entry 2). Increasing the amount of T_3_P^®^ for one equivalent, resulted in a similar conversion (71%) after 30 min (Table 1, Entry 3). Using one equivalent of T_3_P^®^ and applying a reaction time of 1 h, the reaction was complete, and phosphonate **3** was formed in a ratio of 100% (Table 1, Entry 4).

Our aim was also to accomplish the synthesis of dibutyl (2-phenyl-3-(*p*-tolyl)-1,2-dihydroisoquinolin-1-yl)phosphonate (**4**); therefore, the three-component reaction was investigated in the presence of various copper catalysts (Table 1, Entries 5–9). Carrying out the condensation using 5 mol% of CuSO_4_·5H_2_O at 60 °C for 1 h under solvent-free conditions, the cyclic phosphonate (**4**) was the main product (68%); however, 5% of phosphonate **3** and 27% of unreacted dibutyl phosphite (**C**) was also detected in the reaction mixture (Table 1, Entry 5). Repeating this reaction in acetonitrile, the condensation was much more efficient since 86% of cyclic phosphonate (**4**) was formed (Table 1, Entry 6). Applying CuI, CuBr, or CuCl as catalysts, the results obtained were somewhat similar, but CuCl was proved to be the most effective (Table 1, Entries 7–9). To improve the conversion, an experiment was performed in the presence of 10 mol% of CuCl at 60 °C for 1 h, and another at a higher temperature of 80 °C for 1 h, as well as a third at 60 °C for 1.5 h (Table 1, Entries 10–12). It was found that the conversion of each reaction did not change significantly. Next, the condensation was carried out using a small excess of aniline in the presence of 5 mol% of CuCl at 60 °C for 1 h, and the reaction was almost complete (Table 1, Entry 12). Repeating the condensation with 1.2 equivalents of acetylene and aniline under the same conditions, 100% of cyclic phosphonate (**4**) was formed (Table 1, Entry 14).

**Figure 2:**
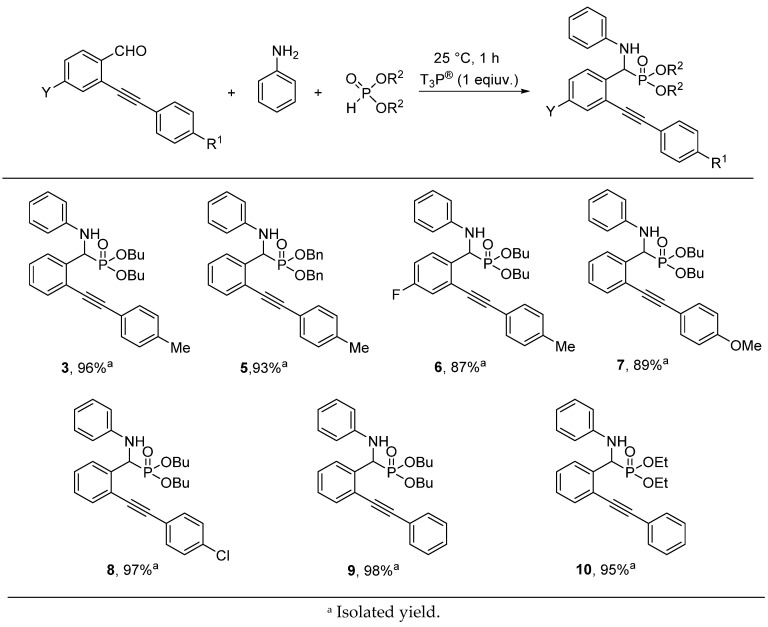
T_3_P^®^-mediated condensation of 2-alkynylbenzaldehydes, aniline and dialkyl phosphites.

In the next round, the T_3_P^®^-promoted Kabachnik–Fields reaction was carried out starting from various 2-alkynylbenzaldehydes, aniline, and dialkyl phosphites under the optimized conditions (1 equiv of T_3_P^®^, 25 °C, 1 h) (Figure 2). The dibutyl ((phenylamino)(2-(*p*-tolylethynyl)phenyl)methyl)phosphonate (**3**) was isolated from the experiment marked by Table 1, Entry 4 in a yield of 96% after the extraction. Performing the condensation of (2-(*p*-tolylethynyl)benzaldehyde with aniline and dibenzyl phosphite, compound **5** was synthesized in a yield of 93%. The three-component reaction of aniline and dibutyl phosphite was also carried out with 4-fluoro-2-(*p*-tolylethynyl)-, 2-((4-methoxyphenyl)ethynyl)-, and 2-((4-chlorophenyl)ethynyl)benzaldehyde, as well as with 2-(phenylethynyl)benzaldehyde, and the corresponding α-aminophosphonates (**6**–**9**) were obtained in yields of 87–98%. The reactions of 2-alkynylbenzaldehydes containing an electron donating group, such as methyl or methoxy group, on the phenyl ring, resulted in slightly lower yields (87% or 89%, respectively). In contrast, α-amino (2-alkynylphenyl)-methylphosphonates bearing a 4-chloro substituent on the phenyl ring (**9**) or the unsubstituted derivatives (**10**) were isolated in excellent yields. Finally, the condensation of 2-(phenylethynyl)benzaldehyde and aniline was carried out with diethyl phosphite, and the result obtained was similar to that of the reaction performed with dibutyl phosphite.

In contrast with previous reports, where magnesium perchlorate [23] or Lewis acids [24] were used as catalysts, the T_3_P^®^-mediated method developed is a new approach for the synthesis of α-amino (2-alkynylphenyl)-methylphosphonates, which applies green, low toxic additive, and milder reaction conditions (25 °C, 1 h). Altogether seven new derivatives were prepared in high yields and characterized by ^31^P, ^1^H, and ^13^C NMR spectroscopy, as well as by HRMS. (Copies of ^31^P, ^1^H, and ^13^C NMR spectra for all compounds synthesized are presented in the Supplementary Materials.)

**Scheme 2:**
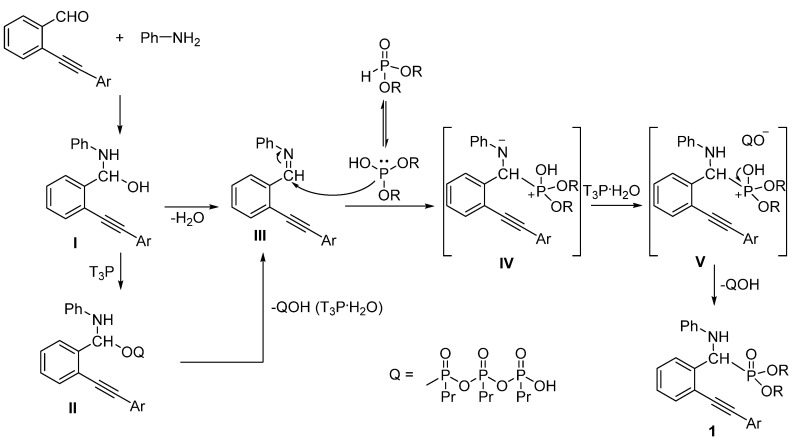
Proposed mechanism for the T_3_P^®^-mediated synthesis of α-amino (2-alkynylphenyl)-methylphosphonates.

The formation of α-amino (2-alkynylphenyl)-methylphosphonates by the T_3_P^®^-promoted Kabachnik–Fields reaction can be explained by the proposed mechanism shown in Scheme 2. First, by the reaction of 2-alkynylbenzaldehyde and aniline, imine **III** is formed via adduct **I**. This condensation may be promoted by T_3_P^®^ to afford imine **III** along with tripropyl triphosphonic acid (QOH) as the by-product. The dehydration may take place via adduct **II**. In the next step, imine **III** reacts with the dialkyl phosphite in a nucleophilic addition, and after a protonation by T_3_P^.^H_2_O, the phosphonium salt **V** formed is stabilized by an Arbuzov fission to furnish α-amino (2-alkynylphenyl)-methylphosphonates (**1**) and the T_3_P^.^H_2_O by-product.

**Figure 3:**
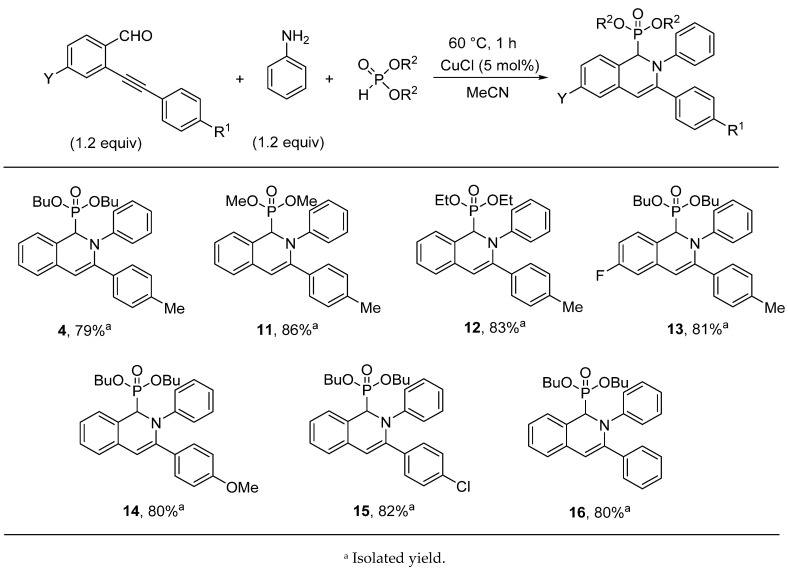
CuCl-catalyzed condensation of 2-alkynylbenzaldehydes, aniline, and dialkyl phosphites.

In the next series of experiments, the CuCl-catalyzed special Kabachnik–Fields reaction of 2-alkynylbenzaldehydes, aniline, and dialkyl phosphites was extended using the optimized conditions (5 mol% CuCl, MeCN, 60 °C, 1 h) (Figure 3). The dibutyl (2-phenyl-3-(*p*-tolyl)-1,2-dihydroisoquinolin-1-yl)phosphonate (**4**) was isolated from the experiment marked by Table 1, Entry 14 in a yield of 79% after column chromatography. The condensation of (2-(*p*-tolylethynyl)benzaldehyde and aniline was also carried out with dimethyl or diethyl phosphite as the P-reagent, and the (2-phenyl-3-(*p*-tolyl)-1,2-dihydroisoquinolin-1-yl)phosphonates (**11** and **12**) were obtained in yields of 86% and 83%, respectively. Performing the three-component reaction of aniline and dibutyl phosphite with 4-fluoro-2-(*p*-tolylethynyl)-, 2-((4-methoxyphenyl)ethynyl)-, and 2-((4-chlorophenyl)ethynyl)benzaldehyde, as well as with 2-(phenylethynyl)benzaldehyde, the corresponding dialkyl (2-phenyl-3-aryl-1,2-dihydroisoquinolin-1-yl)phosphonates (**13**–**16**) were isolated in yields of 80–82% after column chromatography.

In contrast with previous reports, which were detailed in the Introduction section [23,24,25,26], our CuCl-catalyzed approach for the preparation of 2,3-disubstituted-1,2-dihydroisoquinolin-1-ylphosphonates utilizes a small excess of 2-alkynylbenzaldehydes and aniline, less of an amount of catalyst, acetonitrile solvent, a reaction temperature of 60 °C, and a shorter reaction time (1 h). Altogether seven new derivatives were synthesized in good yields (79–86%) and were characterized by ^31^P, ^1^H, and ^13^C NMR spectroscopy, as well as by HRMS. (Copies of ^31^P, ^1^H, and ^13^C NMR spectra for all compounds synthesized are presented in the Supplementary Materials.)

**Scheme 3:**
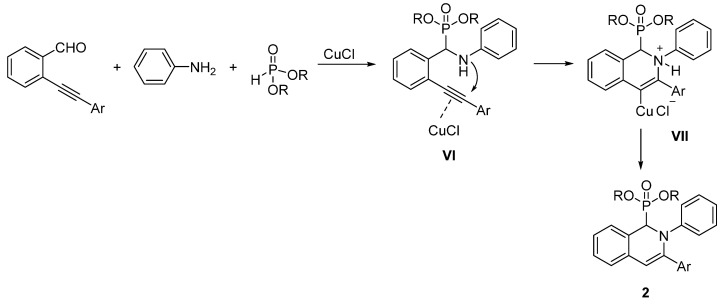
Proposed mechanism for the CuCl-catalyzed synthesis of 1,2-dihydroisoquinolin-1-ylphosphonates.

The formation of 1,2-dihydroisoquinolin-1-ylphosphonates (**2**) by the CuCl-catalyzed three-component reaction can be explained by the mechanism shown in Scheme 3. First, by the Kabachnik–Fields reaction of 2-alkynylbenzaldehyde, aniline, and dialkyl phosphite, α-aminophosphonate **VI** is formed. Then, the CuCl catalyst activates the triple bond for the intramolecular nucleophile attack of the amino group. This ring closure step results in an organocopper cyclic ammonium salt **VII**, which is stabilized by a deprotonation/protonation step (parallelly losing the CuCl unit) to form the 1,2-dihydroisoquinolin-1-ylphosphonate (**2**).

## 4. Conclusions

In summary, we developed a novel approach for the synthesis of new α-amino (2-alkynylphenyl)-methylphosphonates by the T_3_P^®^-mediated three-component reaction of 2-alkynylbenzaldehydes, aniline, and dialkyl phosphites. The method developed has the advantages of the simple operation and mild reaction conditions; furthermore, it does not require a chromatographic separation since the products could be recovered from the reaction mixture by an extraction. Moreover, novel 2,3-disubstituted-1,2-dihydroisoquinolin-1-ylphosphonates were synthesized by the CuCl-catalyzed condensation of the same kinds of starting materials (2-alkynylbenzaldehydes, aniline, and dialkyl phosphites) at 60 °C for a short reaction time (1 h). This approach is faster and cheaper compared with the examples in the literature, where the reactions were complete after 4–6 h using more expensive catalysts. Applying the methods developed, altogether seven α-amino (2-alkynylphenyl)-methylphosphonates and seven 2,3-disubstituted-1,2-dihydroisoquinolin-1-ylphosphonate derivatives were synthesized in good to high yields, and fully characterized, all of them are new compounds.

## Figures and Tables

**Table 1:** Investigation of the condensation of 2-(*p*-tolylethynyl)benzaldehyde, aniline, and dibutyl phosphite.

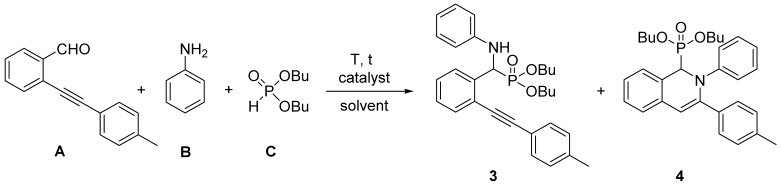								
Entry	A:B:C (Equiv)	Catalyst	Solvent	T (°C)	T (h)	Composition ^a^ (%)		
						C	3	4
1	1:1:1	–	MeCN	60	4	48	50	2
2	1:1:1	0.5 equiv T_3_P^®^	–	25	1	30	70	0
3	1:1:1	1 equiv T_3_P^®^	–	25	0.5	29	71	0
4	1:1:1	1 equiv T_3_P^®^	–	25	1	0	100	0
5	1:1:1	5 mol% CuSO_4_·5H_2_O	–	60	1	27	5	68
6	1:1:1	5 mol% CuSO_4_·5H_2_O	MeCN	60	1	14	0	86
7	1:1:1	5 mol% CuI	MeCN	60	1	12	0	88
8	1:1:1	5 mol% CuBr	MeCN	60	1	13	0	87
9	1:1:1	5 mol% CuCl	MeCN	60	1	9	0	91
10	1:1:1	10 mol% CuCl	MeCN	60	1	9	0	91
11	1:1:1	5 mol% CuCl	MeCN	80	1	8	0	92
12	1:1:1	5 mol% CuCl	MeCN	60	1.5	8	0	92
13	1:1.2:1	5 mol% CuCl	MeCN	60	1	5	0	95
13	1.2:1.2:1	5 mol% CuCl	MeCN	60	1	0	0	100

^a^ Determined by ^31^P NMR.

## Data Availability

Data is contained within the article or Supplementary Materials.

## Supplementary Materials

The following are available online at https://www.mdpi.com/article/10.3390/ma14206015/s1, Copies of ^31^P, ^1^H, and ^13^C NMR spectra for all compounds synthesized are presented.
