# Supplementary material for: Study of the Three-Component Reactions of 2-Alkynylbenzaldehydes, Aniline, and Dialkyl Phosphites—The Significance of the Catalyst System
Source: Materials (Basel). 2021 Oct 13;14(20):6015. doi: 10.3390/ma14206015 (PMC8539604; doi:10.3390/ma14206015)
Supplement: Supplementary file 1 [file materials-14-06015-s001.zip › materials-1395029-supplementary.pdf]

Supporting Information

Study of the Three-Component Reactions of 2-Alkynylbenzaldehydes, Aniline, and Dialkyl Phosphites—The Significance of the Catalyst System

Nóra Popovics-Tóth, Kármen Emőke Szabó and Erika Bálint *

Department of Organic Chemistry and Technology, Budapest University of Technology and Economics, 1521 Budapest, Hungary; nora.toth@edu.bme.hu (N.P.-T.); szabo.karmen948@gmail.com (K.E.S.)

* Correspondence: balint.erika@vbk.bme.hu; Tel.: +36-1-463-3653

Table of contents

^{31}P NMR, ^{1}H NMR and ^{13}C NMR spectra: S2–S22

^{31}P NMR, ^{1}H NMR and ^{13}C NMR spectra

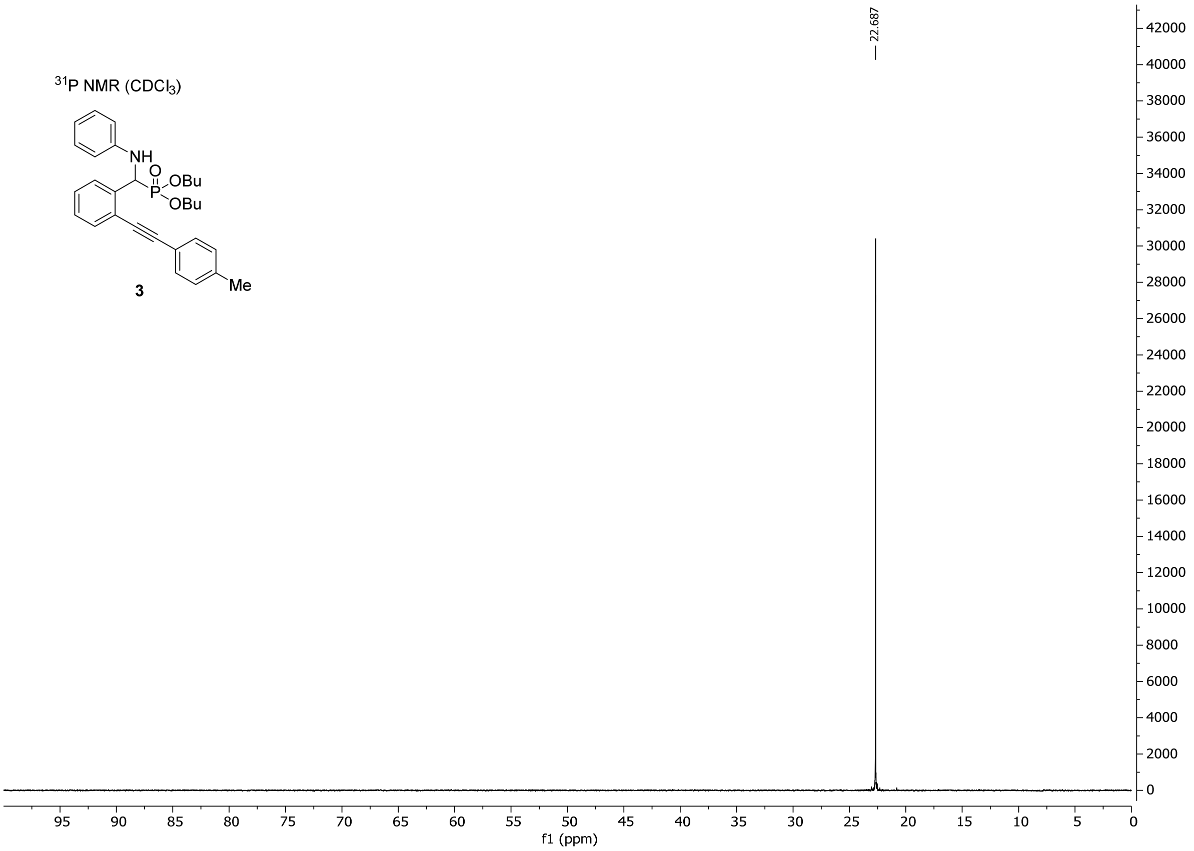

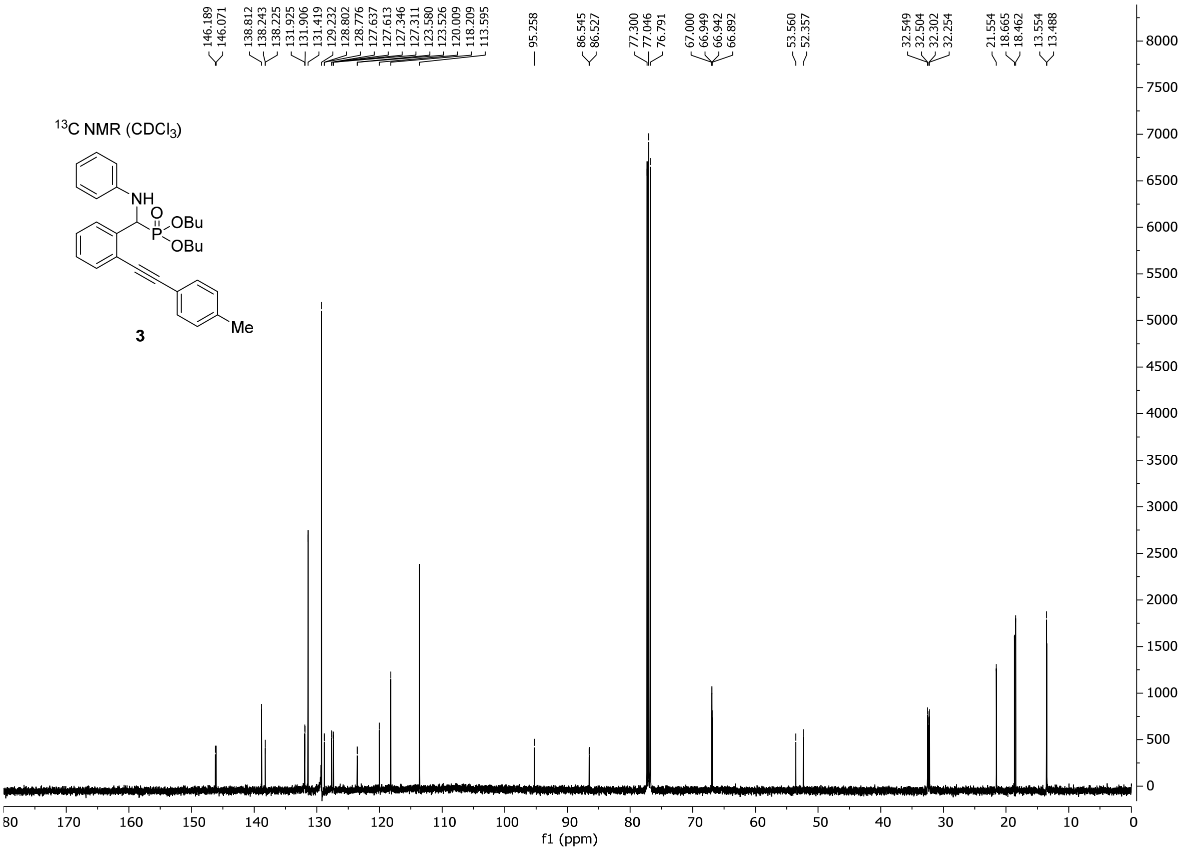

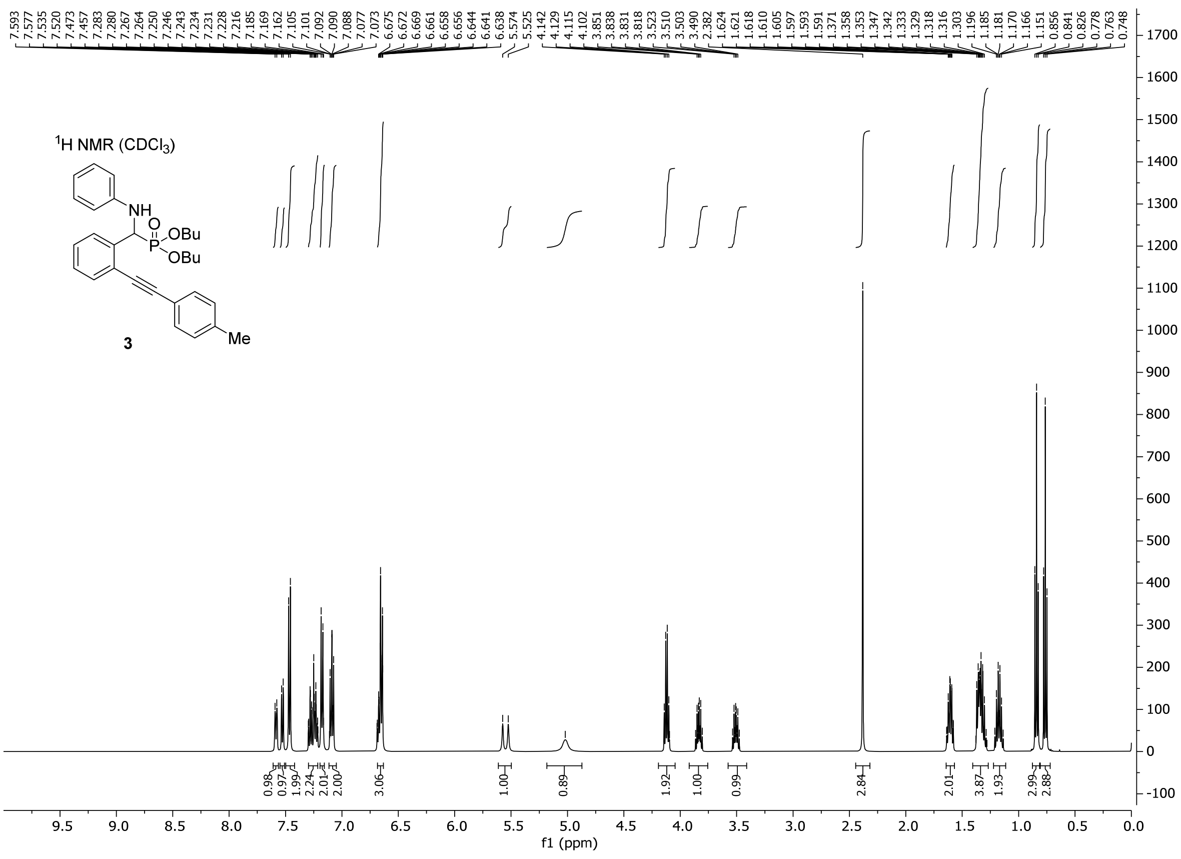
1H NMR (CDCl3)
NH
O
P
OBu
OBu
Me
3
f1 (ppm)

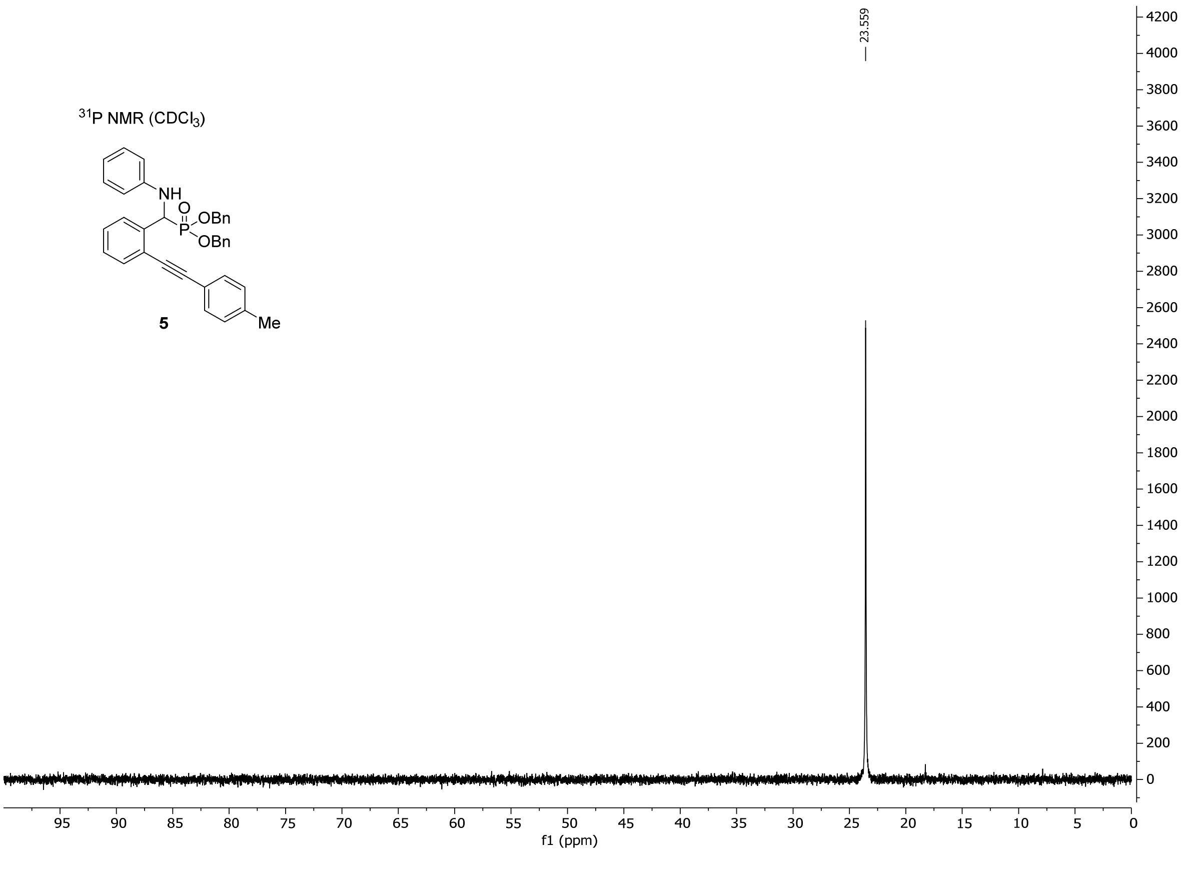
23.559
31P NMR (CDCl3)
NH
O
P
OBn
OBn
Me
5
f1 (ppm)

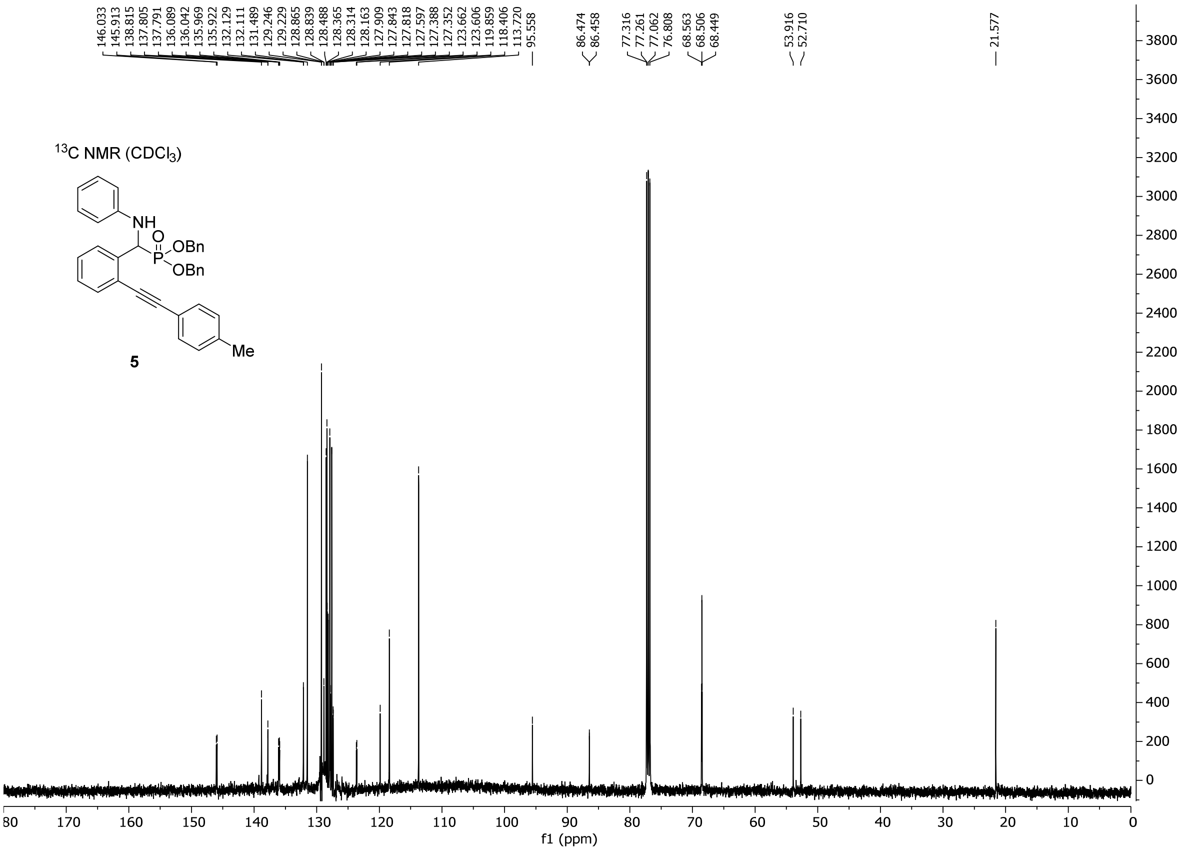
146.033 145.913 138.815 137.805 137.791 136.089 136.042 135.969 135.922 132.129 132.111 131.489 129.246 129.229 128.865 128.839 128.488 128.365 128.314 128.163 127.909 127.843 127.818 127.597 127.388 127.352 123.662 123.606 119.859 118.406 113.720 95.558
86.474 86.458
77.316 77.261 77.062 76.808
68.563 68.506 68.449
53.916 52.710
21.577
13C NMR (CDCl3)
NH
O
P
OBn
OBn
Me
5
f1 (ppm)

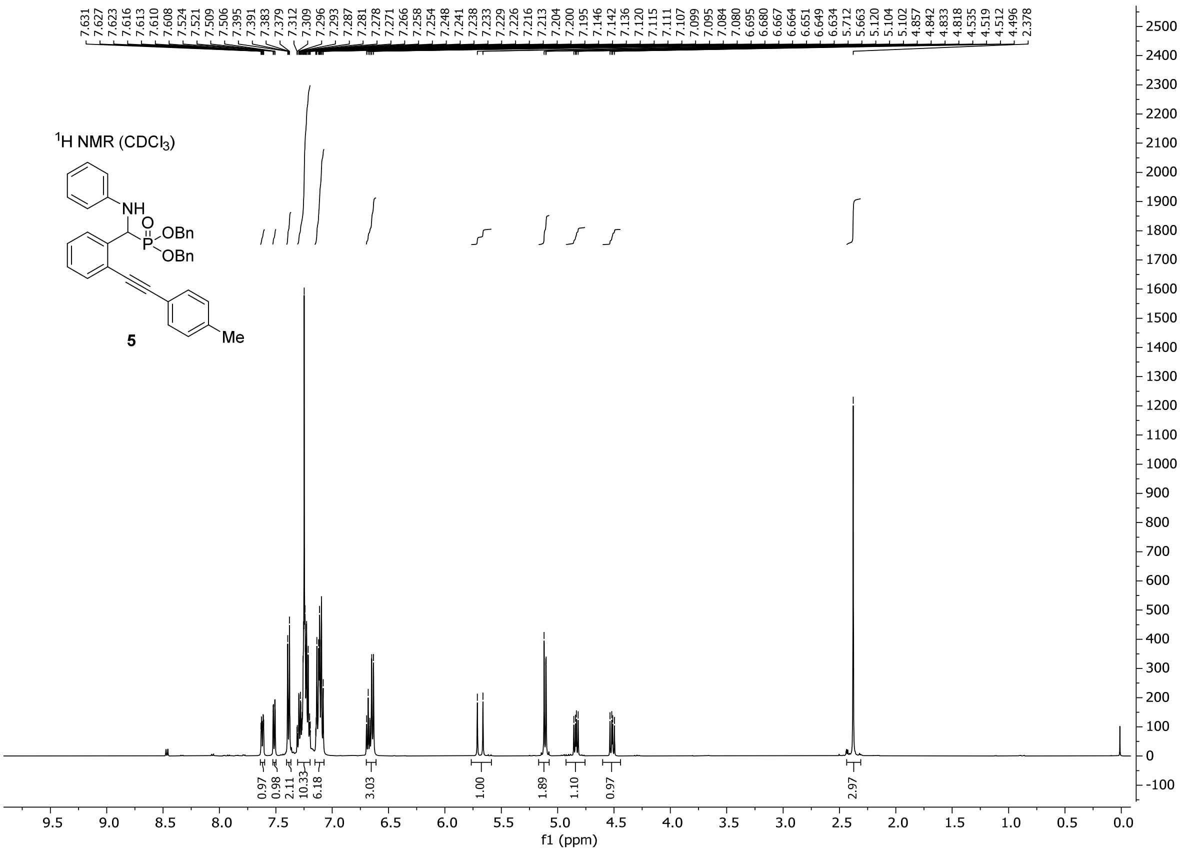
7.631 7.627 7.623 7.616 7.613 7.610 7.608 7.524 7.521 7.509 7.506 7.395 7.391 7.383 7.379 7.312 7.309 7.296 7.293 7.287 7.281 7.278 7.271 7.266 7.258 7.254 7.248 7.241 7.238 7.233 7.229 7.226 7.216 7.213 7.204 7.200 7.195 7.146 7.142 7.136 7.120 7.115 7.111 7.107 7.099 7.095 7.084 7.080 6.695 6.680 6.667 6.664 6.651 6.649 6.634 5.712 5.663 5.120 5.104 5.102 4.857 4.842 4.833 4.818 4.535 4.519 4.512 4.496 2.378
1H NMR (CDCl3)
NH
O
P
OBn
OBn
Me
5
0.97 0.98 2.11 10.33 6.18 3.03 1.00 1.89 1.10 0.97 2.97
f1 (ppm)

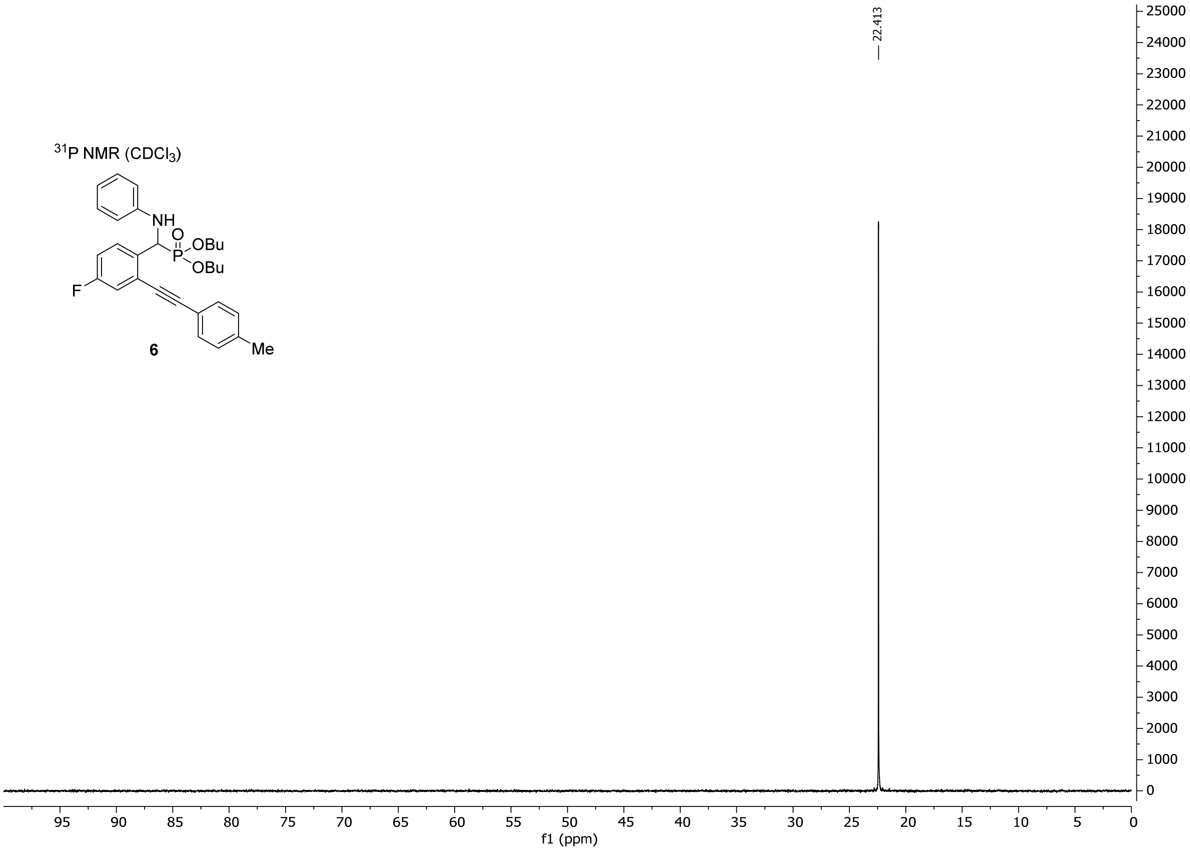
^{31}P NMR (CDCl$_3$)
NH
O
P
OBu
OBu
F
Me
6
22.413
f1 (ppm)

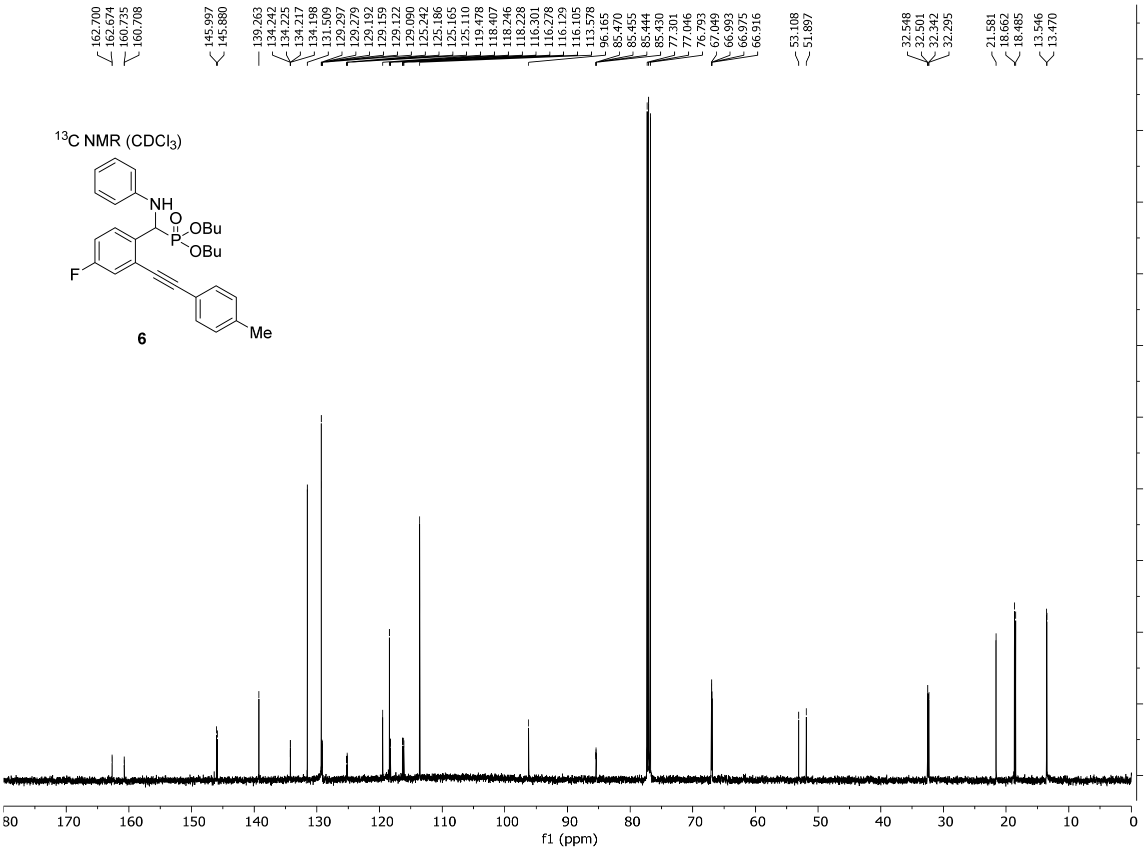
^{13}C NMR (CDCl$_3$)
NH
O
P
OBu
OBu
F
Me
6
162.700
162.674
160.735
160.708
145.997
145.880
139.263
134.242
134.225
134.217
134.198
131.509
129.297
129.279
129.192
129.159
129.122
129.090
125.242
125.186
125.165
125.110
119.478
118.407
118.246
118.228
116.301
116.278
116.129
116.105
113.578
96.165
85.470
85.455
85.444
85.430
77.301
77.046
76.793
67.049
66.993
66.975
66.916
53.108
51.897
32.548
32.501
32.342
32.295
21.581
18.662
18.485
13.546
13.470
f1 (ppm)

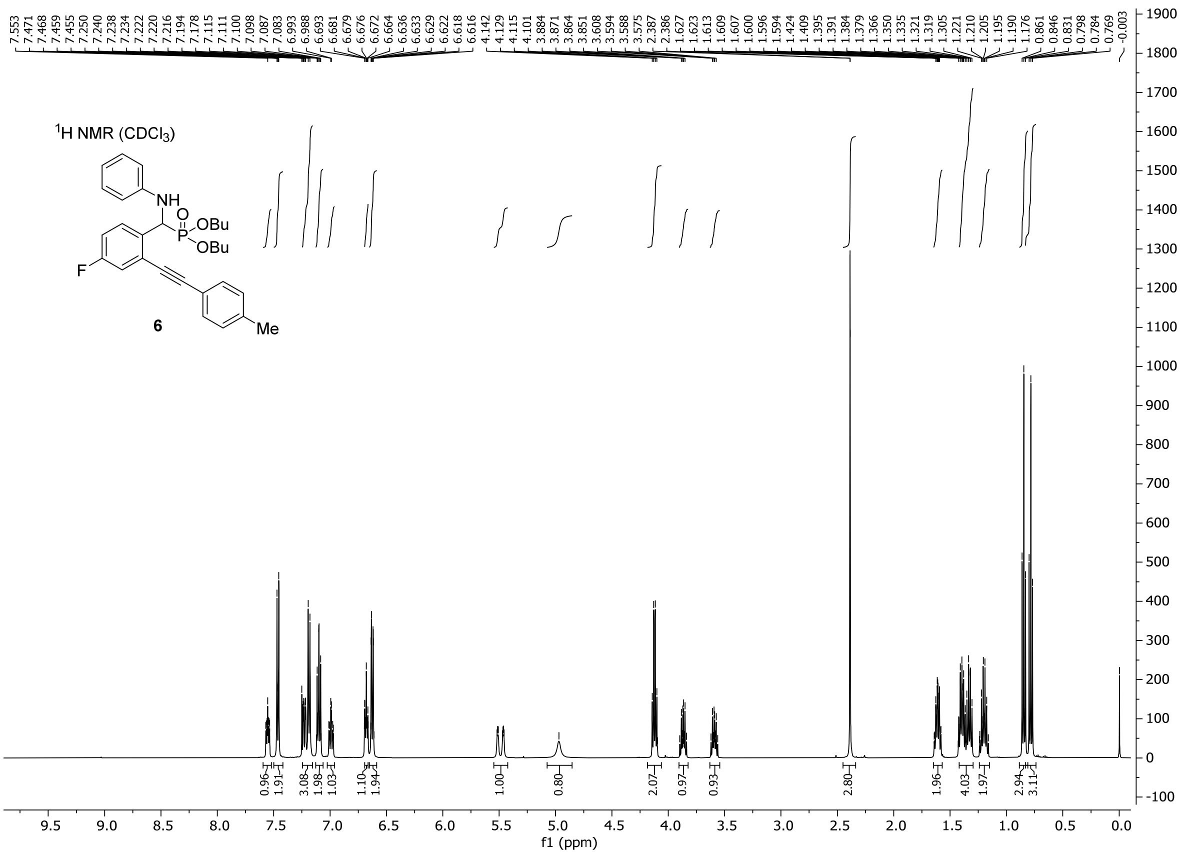

^{1}H NMR (CDCl$_3$)
NH
OBu
OBu
F
Me
6
f1 (ppm)

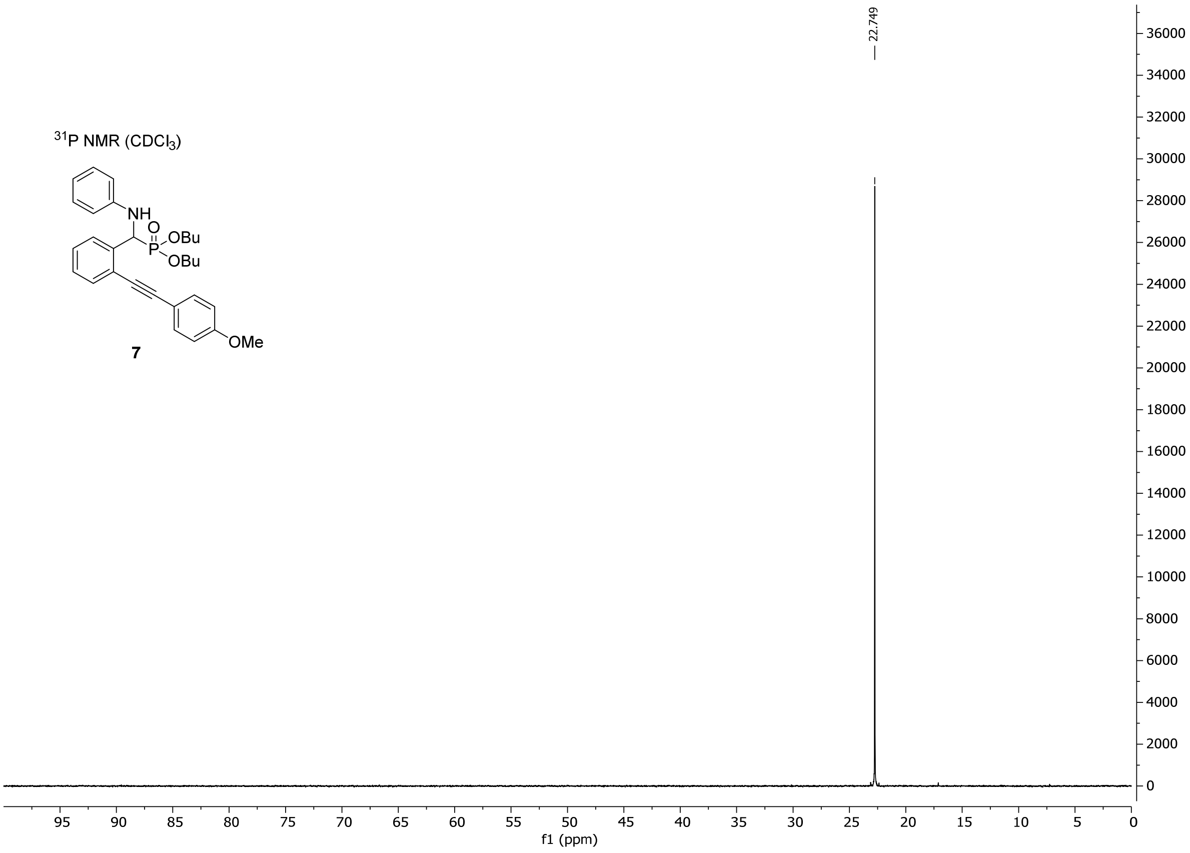

22.749
^{31}P NMR (CDCl$_3$)
NH
OBu
OBu
OMe
7
f1 (ppm)

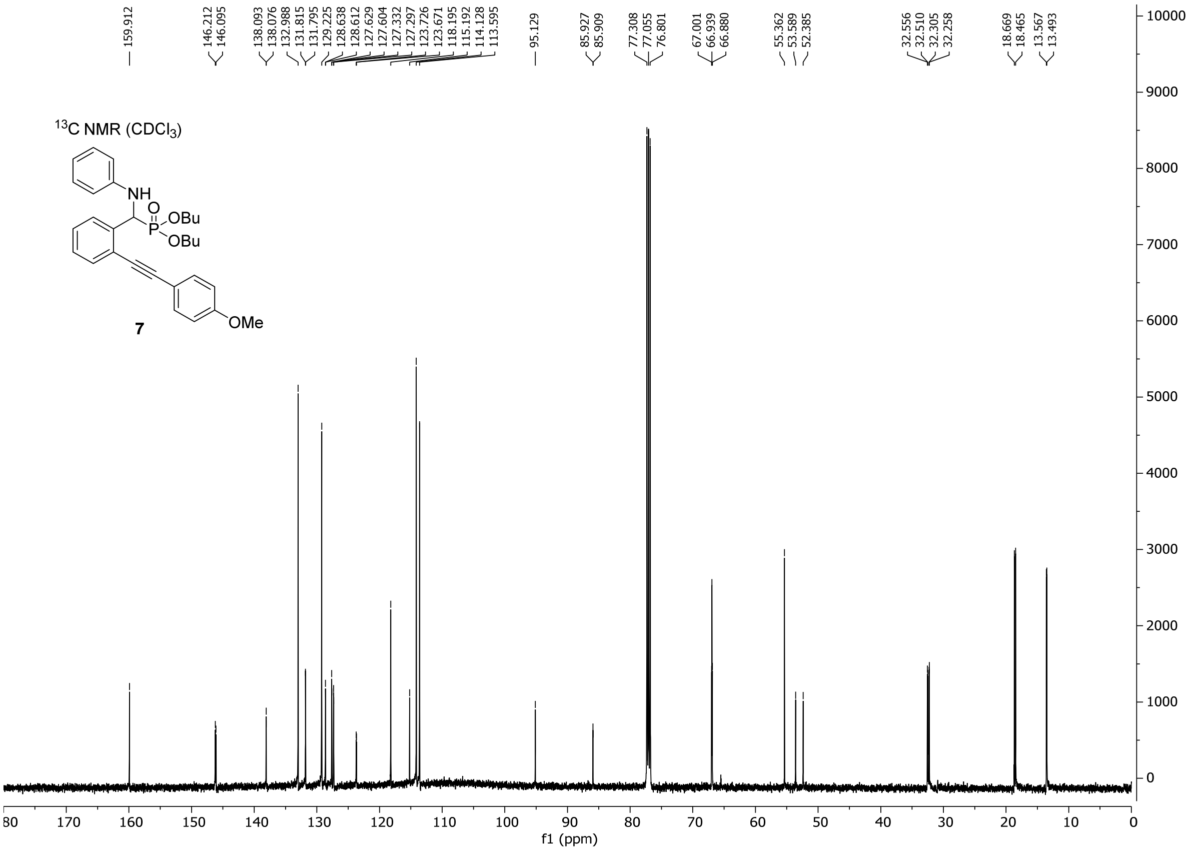

159.912
146.212
146.095
138.093
138.076
132.988
131.815
131.795
129.225
128.638
128.612
127.629
127.604
127.332
127.297
123.726
123.671
118.195
115.192
114.128
113.595
95.129
85.927
85.909
77.308
77.055
76.801
67.001
66.939
66.880
55.362
53.589
52.385
32.556
32.510
32.305
32.258
18.669
18.465
13.567
13.493
13C NMR (CDCl3)
NH
O
P
OBu
OBu
OMe
7
f1 (ppm)

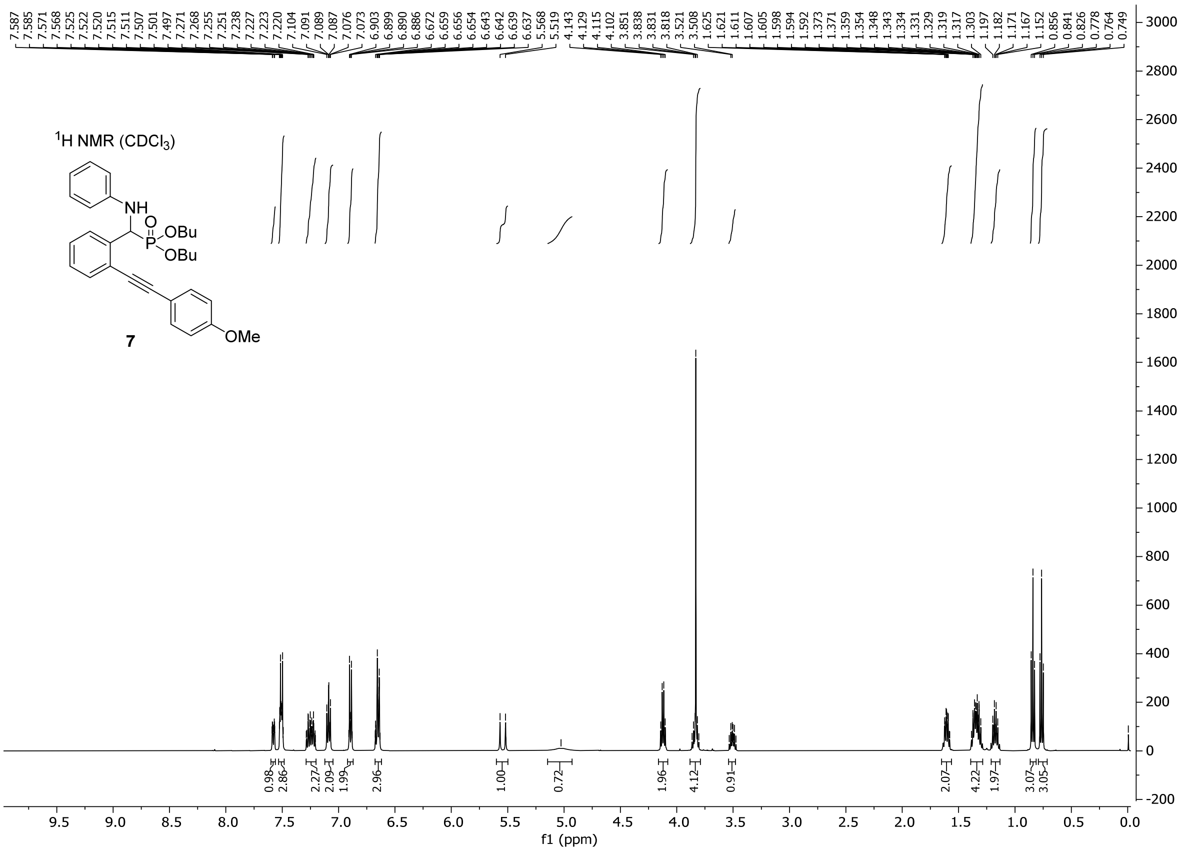

1H NMR (CDCl3)
NH
O
P
OBu
OBu
OMe
7
0.98
2.86
2.27
2.09
1.99
2.96
1.00
0.72
1.96
4.12
0.91
2.07
4.22
1.97
3.07
3.05
f1 (ppm)

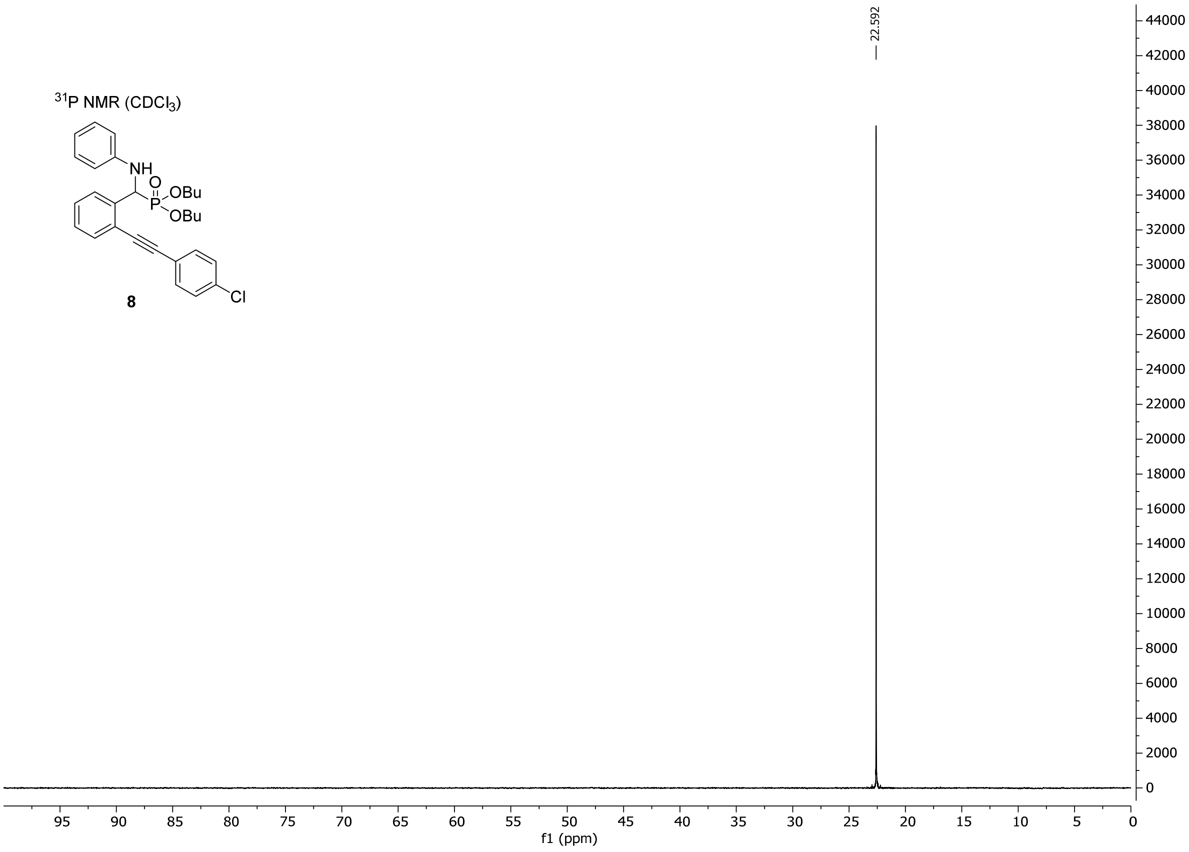

^{31}P NMR (CDCl$_3$)
NH
O
P
OBu
OBu
Cl
8
22.592
f1 (ppm)

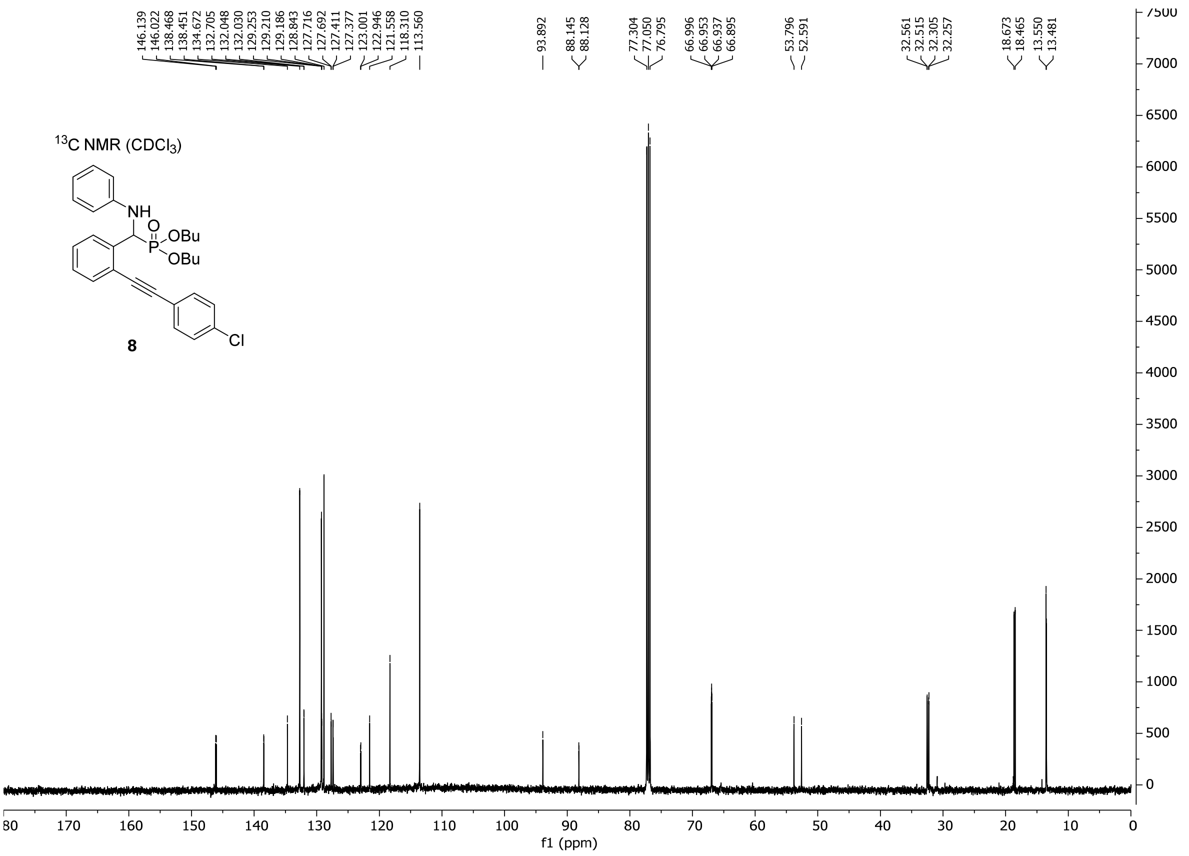

^{13}C NMR (CDCl$_3$)
NH
O
P
OBu
OBu
Cl
8
146.139
146.022
138.468
138.451
134.672
132.705
132.048
132.030
129.253
129.210
129.186
128.843
127.716
127.692
127.411
127.377
123.001
122.946
121.558
118.310
113.560
93.892
88.145
88.128
77.304
77.050
76.795
66.996
66.953
66.937
66.895
53.796
52.591
32.561
32.515
32.305
32.257
18.673
18.465
13.550
13.481
f1 (ppm)

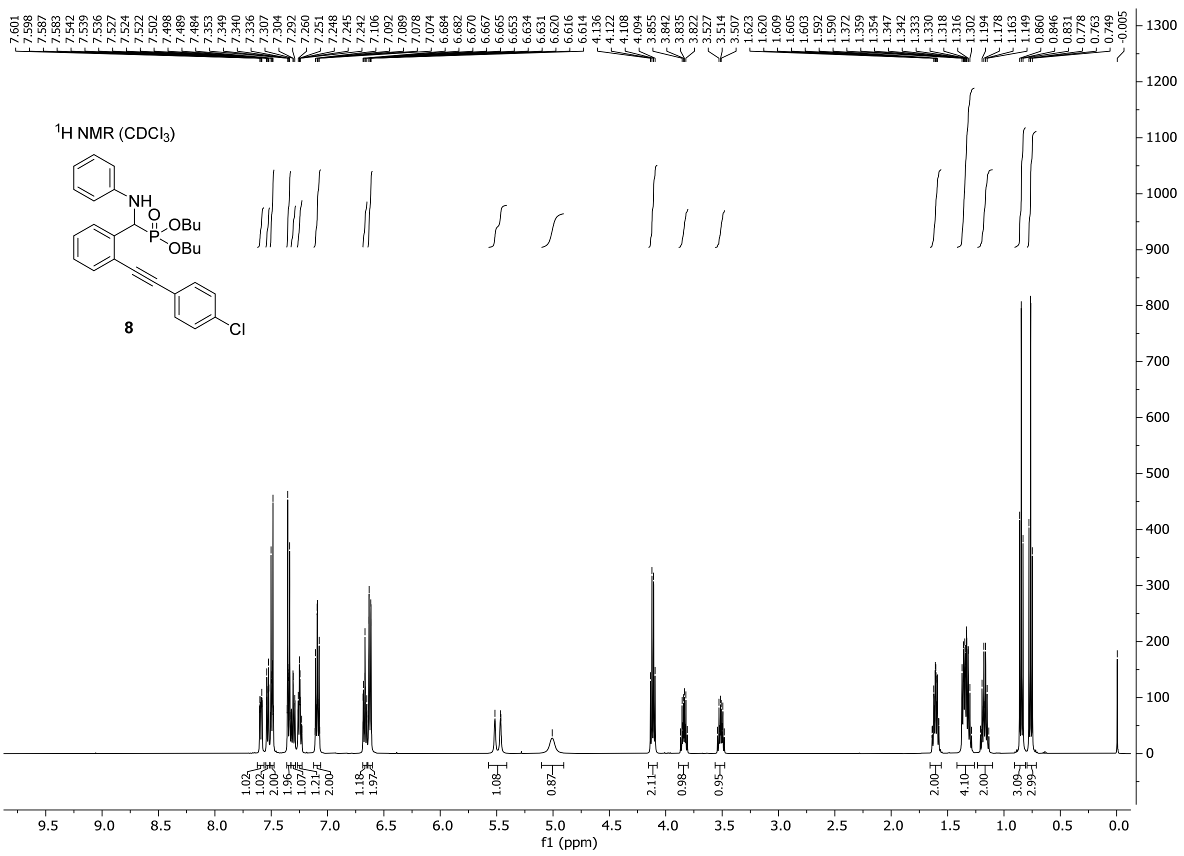
1H NMR (CDCl3)
8
f1 (ppm)

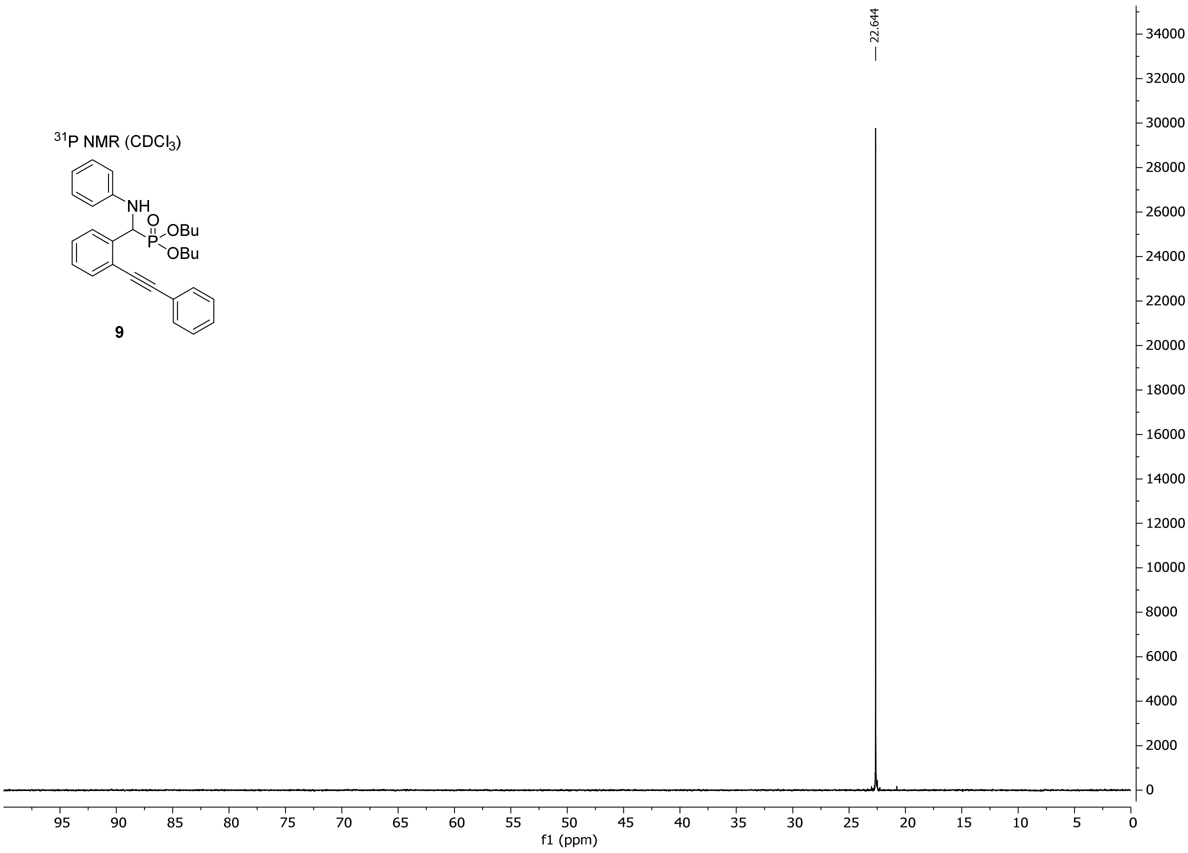
31P NMR (CDCl3)
9
22.644
f1 (ppm)

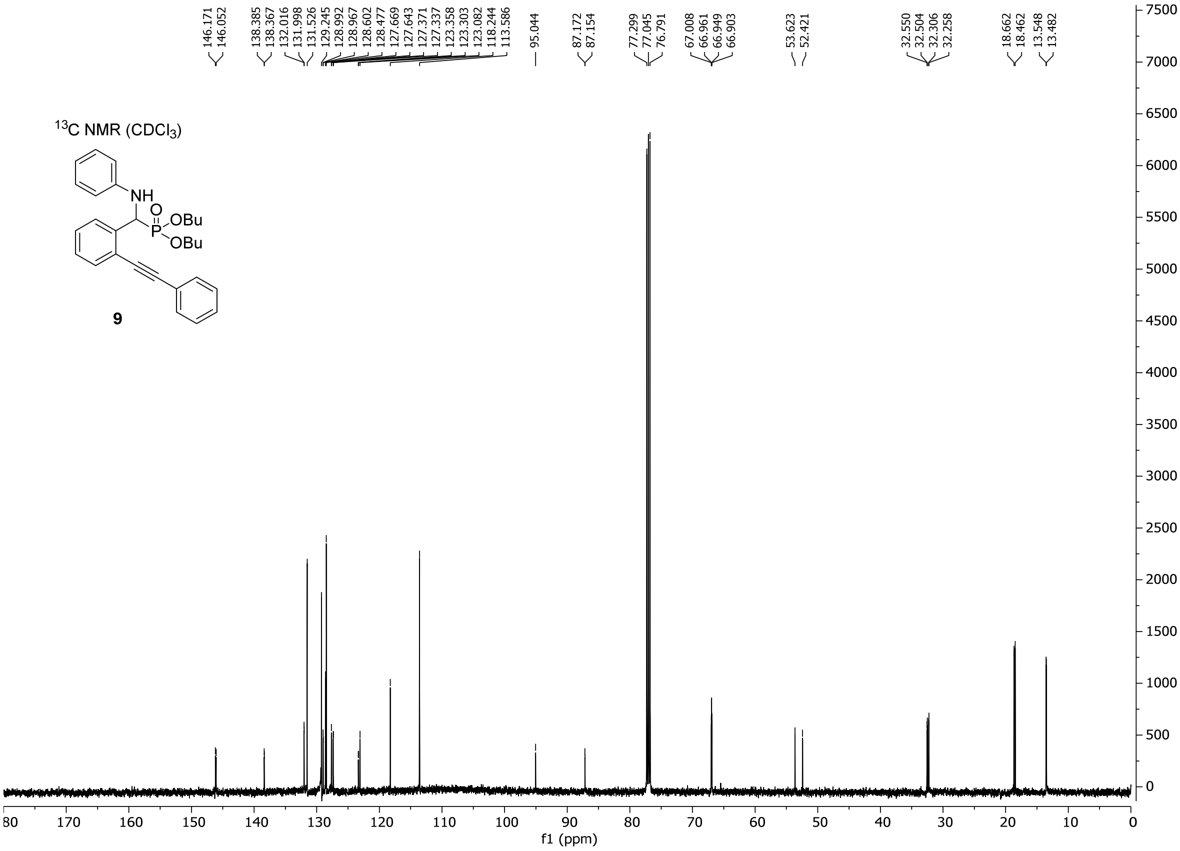
146.171
146.052
138.385
138.367
132.016
131.998
131.526
129.245
128.992
128.967
128.602
128.477
127.669
127.643
127.371
127.337
123.358
123.303
123.082
118.244
113.586
95.044
87.172
87.154
77.299
77.045
76.791
67.008
66.961
66.949
66.903
53.623
52.421
32.550
32.504
32.306
32.258
18.662
18.462
13.548
13.482
^{13}C NMR (CDCl$_3$)
NH
O
P
OBu
OBu
9
f1 (ppm)

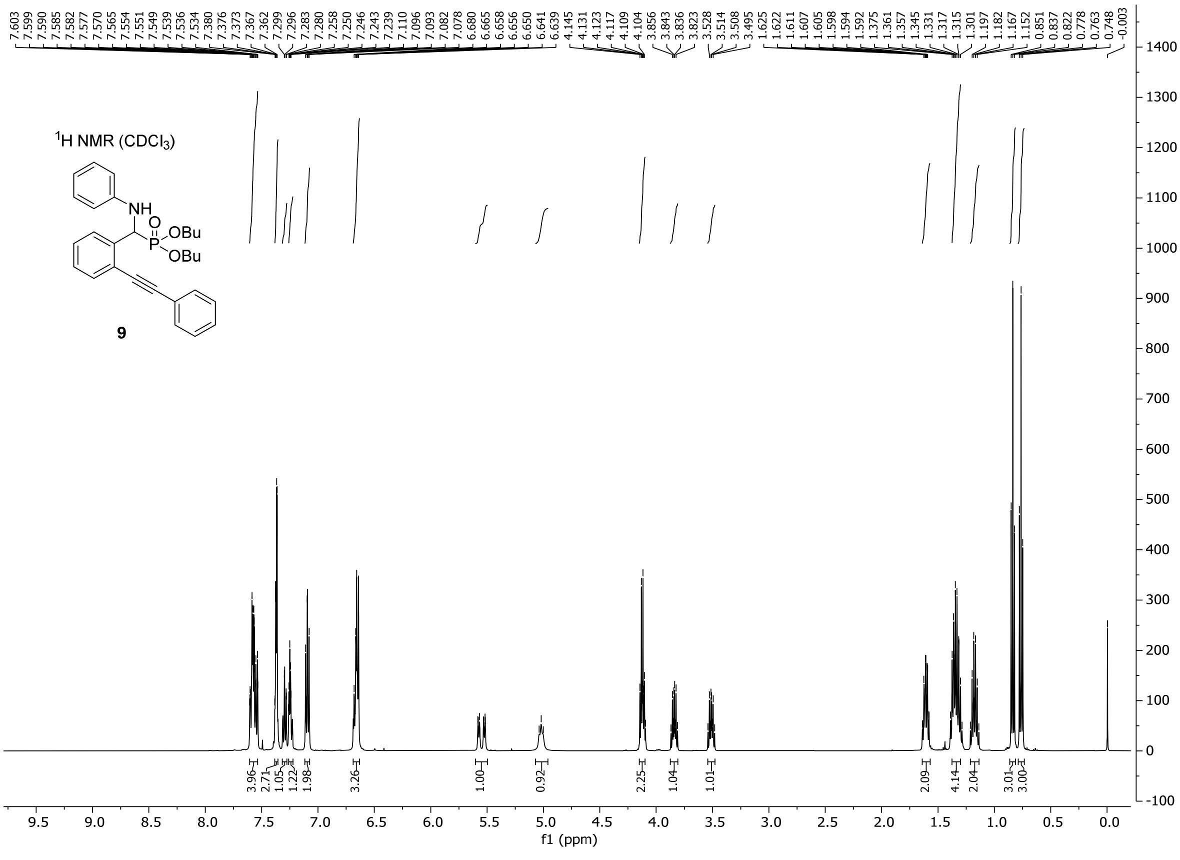
7.603
7.599
7.590
7.585
7.582
7.577
7.570
7.565
7.554
7.551
7.549
7.539
7.536
7.534
7.380
7.376
7.373
7.367
7.362
7.299
7.296
7.283
7.280
7.258
7.250
7.246
7.243
7.239
7.110
7.096
7.093
7.082
7.078
6.680
6.665
6.658
6.656
6.650
6.641
6.639
4.145
4.131
4.123
4.117
4.109
4.104
3.856
3.843
3.836
3.823
3.528
3.514
3.508
3.495
1.625
1.622
1.611
1.607
1.605
1.598
1.594
1.592
1.375
1.361
1.357
1.345
1.331
1.317
1.315
1.301
1.197
1.182
1.167
1.152
0.851
0.837
0.822
0.778
0.763
0.748
-0.003
^{1}H NMR (CDCl$_3$)
NH
O
P
OBu
OBu
9
3.96
2.71
1.05
1.22
1.98
3.26
1.00
0.92
2.25
1.04
1.01
2.09
4.14
2.04
3.01
3.00
f1 (ppm)

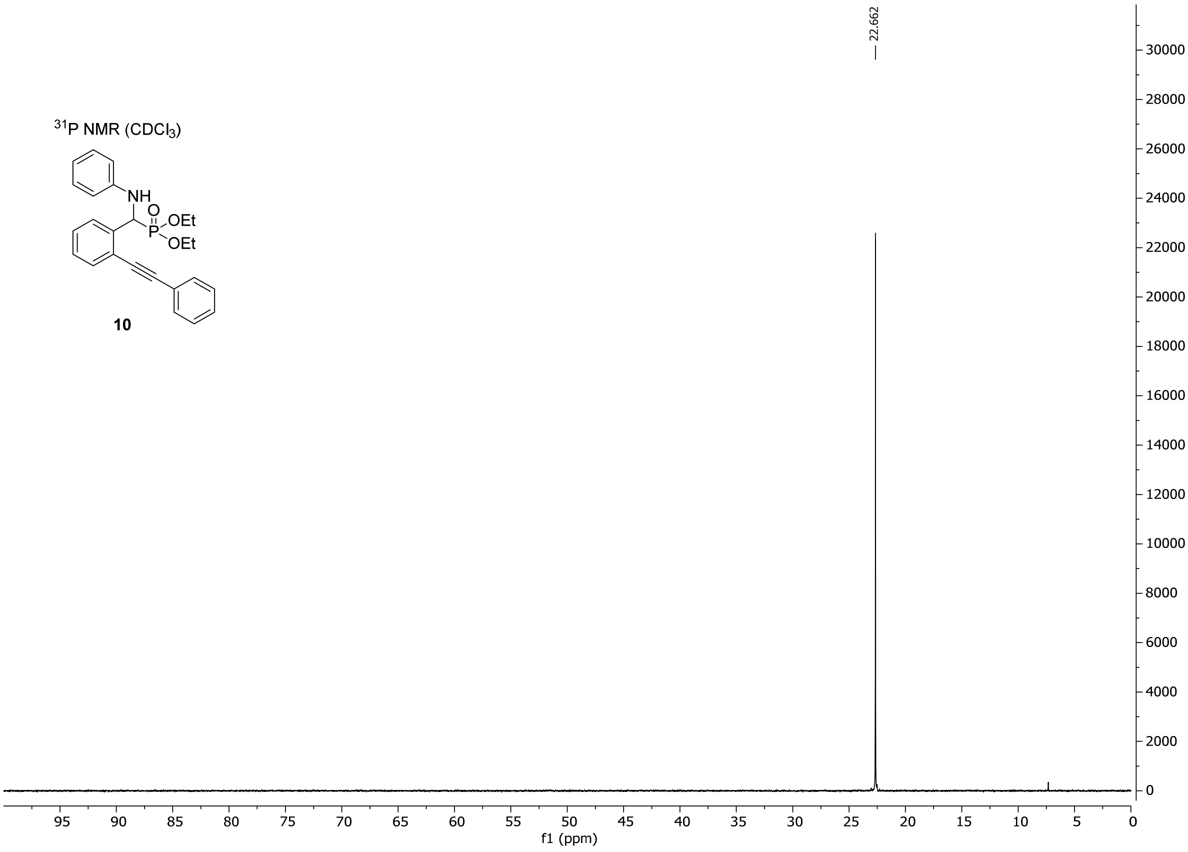
31P NMR (CDCl3)
NH
O
P
OEt
OEt
10
22.662
f1 (ppm)

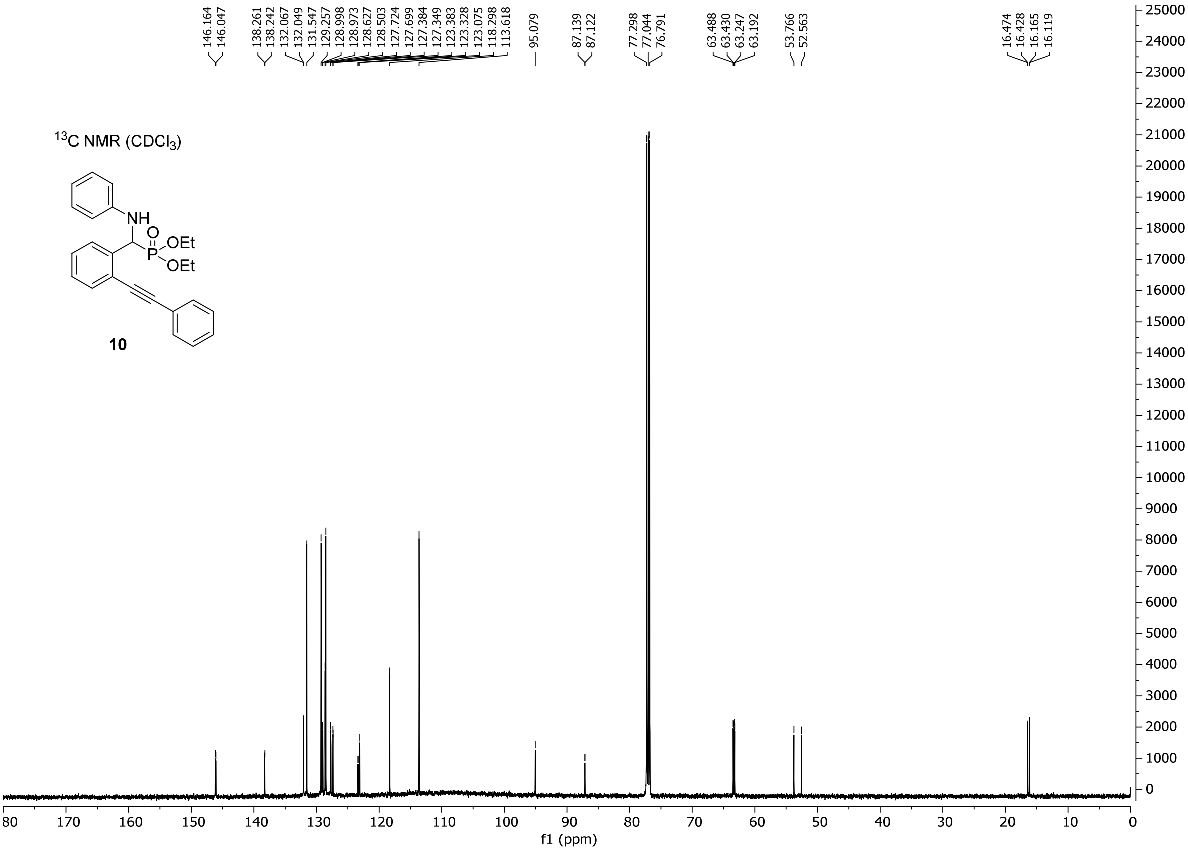
13C NMR (CDCl3)
NH
O
P
OEt
OEt
10
146.164
146.047
138.261
138.242
132.067
132.049
131.547
129.257
128.998
128.973
128.627
128.503
127.724
127.699
127.384
127.349
123.383
123.328
123.075
118.298
113.618
95.079
87.139
87.122
77.298
77.044
76.791
63.488
63.430
63.247
63.192
53.766
52.563
16.474
16.428
16.165
16.119
f1 (ppm)

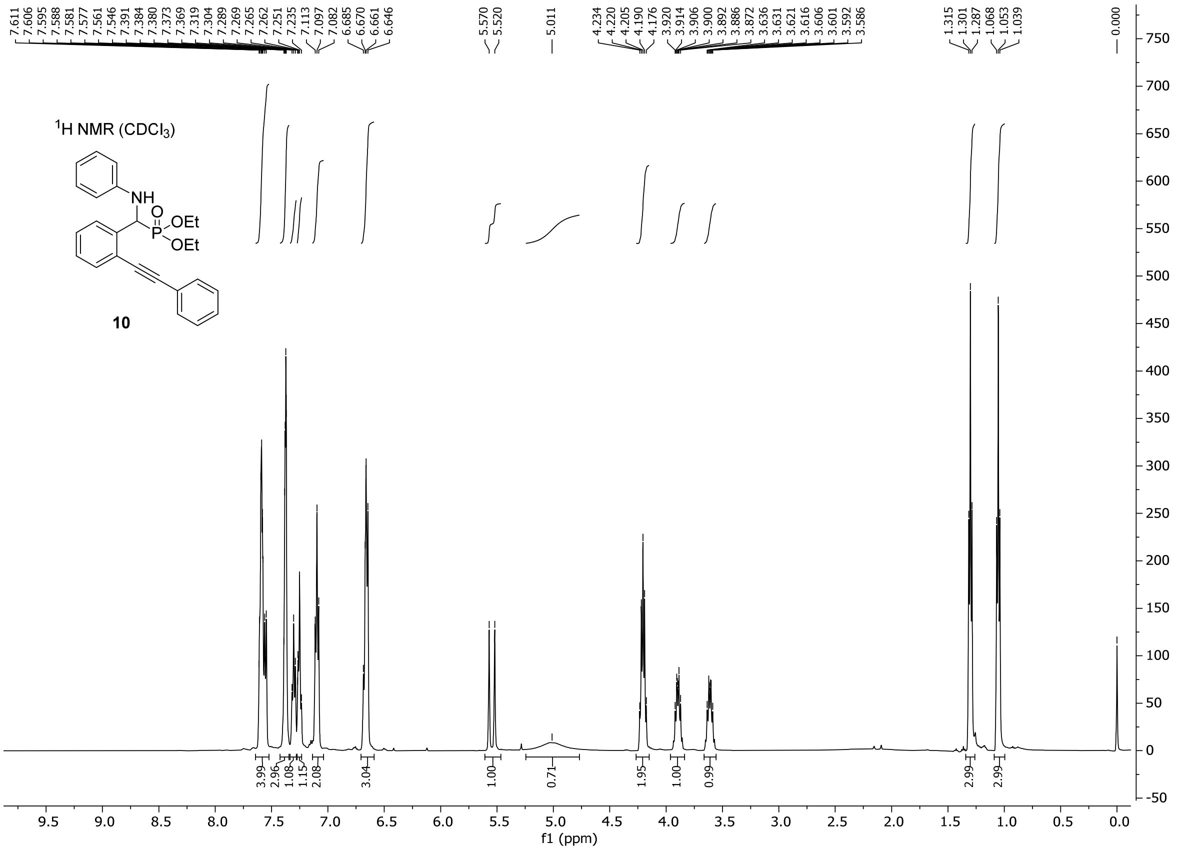

1H NMR (CDCl3)
NH
O
P
OEt
OEt
10
f1 (ppm)

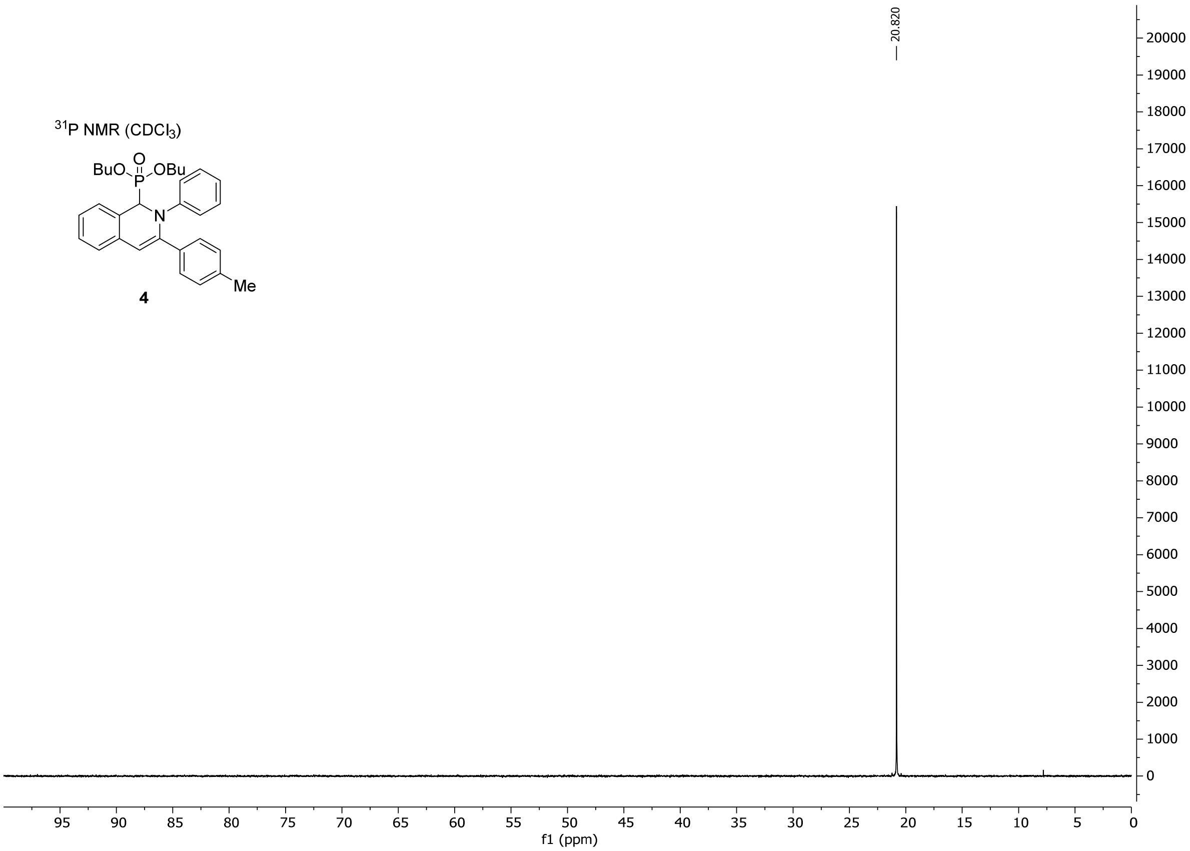

31P NMR (CDCl3)
BuO
O
P
OBu
N
Me
4
20.820
f1 (ppm)

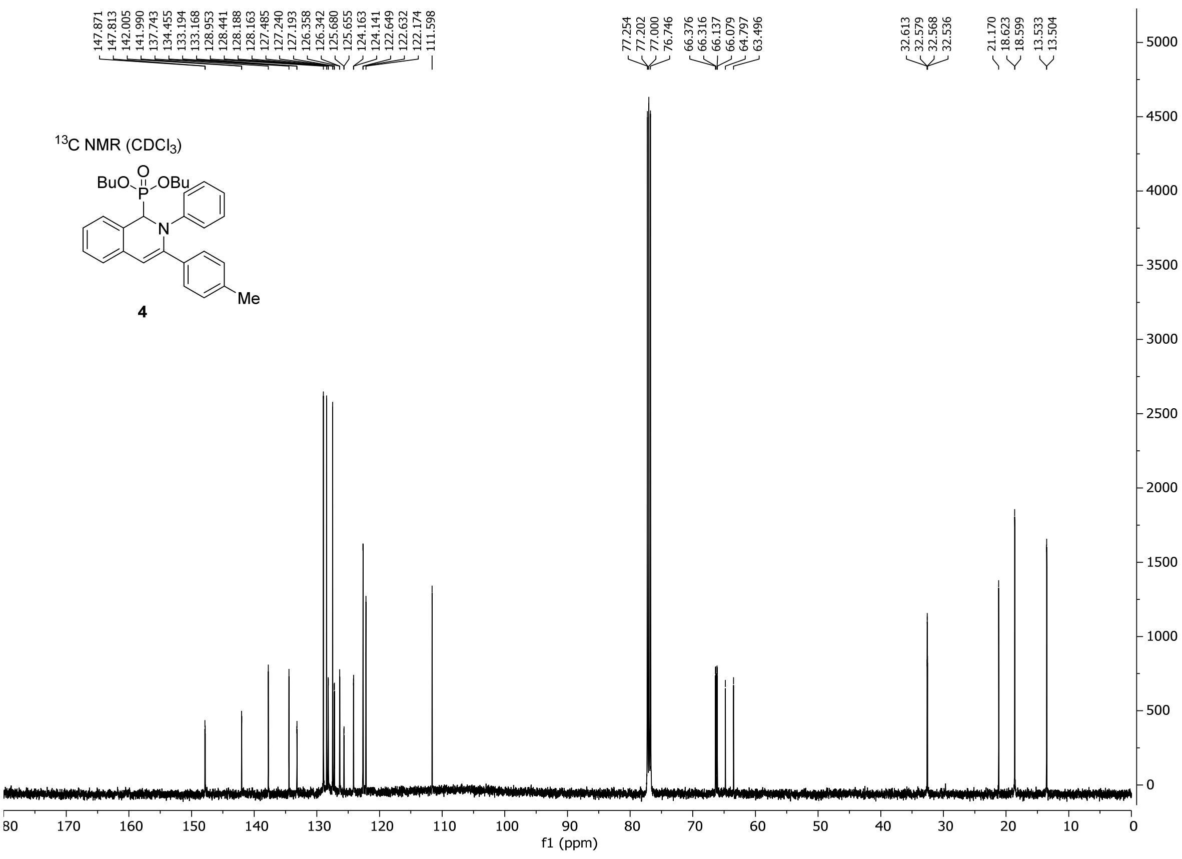
147.871
147.813
142.005
141.990
137.743
134.455
133.194
133.168
128.953
128.441
128.188
128.163
127.485
127.240
127.193
126.358
126.342
125.680
125.655
124.163
124.141
122.649
122.632
122.174
111.598
77.254
77.202
77.000
76.746
66.376
66.316
66.137
66.079
64.797
63.496
32.613
32.579
32.568
32.536
21.170
18.623
18.599
13.533
13.504
13C NMR (CDCl3)
BuO
OBu
O
P
N
Me
4
f1 (ppm)

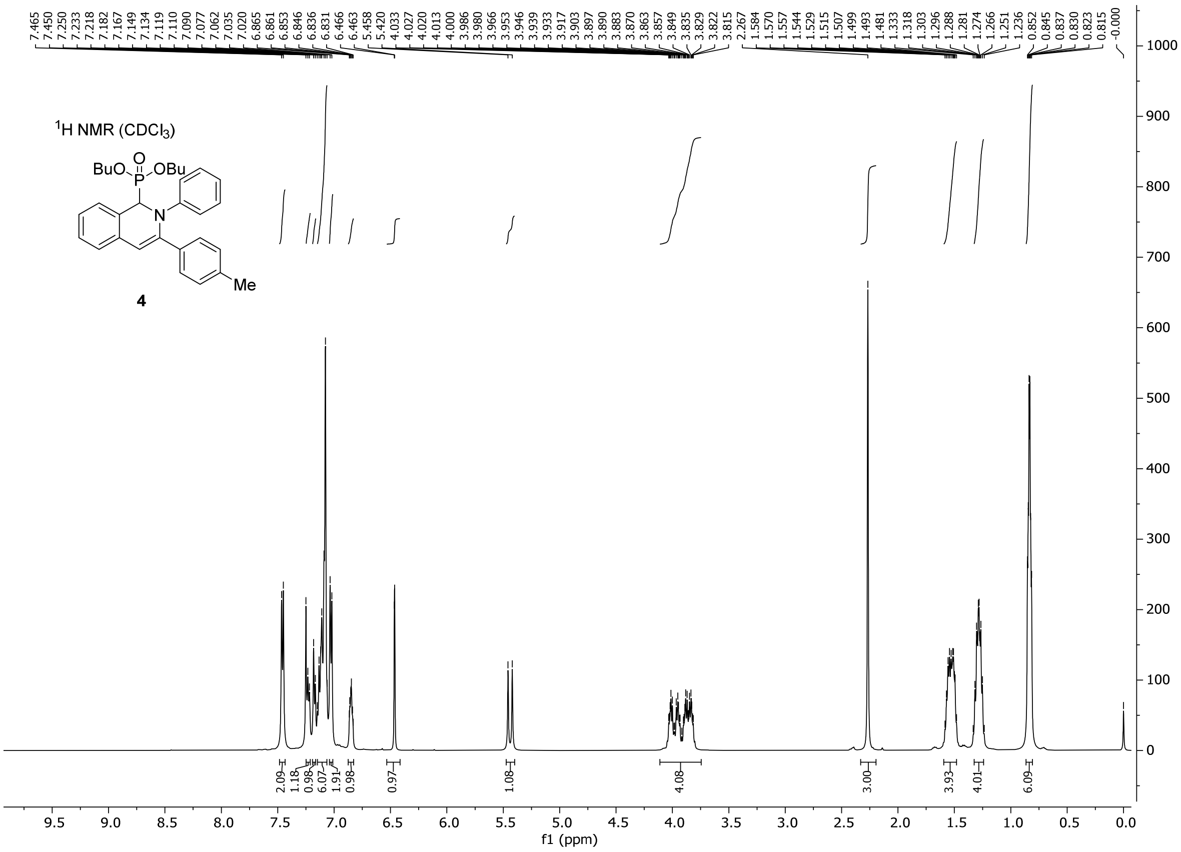
7.465
7.450
7.250
7.233
7.218
7.182
7.167
7.149
7.134
7.119
7.110
7.090
7.077
7.062
7.035
7.020
6.865
6.861
6.853
6.846
6.836
6.831
6.466
6.463
6.458
5.420
4.033
4.027
4.020
4.013
4.000
3.986
3.980
3.966
3.953
3.946
3.939
3.933
3.917
3.903
3.897
3.890
3.883
3.870
3.863
3.857
3.849
3.835
3.829
3.822
3.815
2.267
1.584
1.570
1.557
1.544
1.529
1.515
1.507
1.499
1.493
1.481
1.333
1.318
1.303
1.296
1.288
1.281
1.274
1.266
1.251
1.236
0.852
0.845
0.837
0.830
0.823
0.815
-0.000
1H NMR (CDCl3)
BuO
OBu
O
P
N
Me
4
2.09
1.18
0.98
6.07
1.91
0.98
0.97
1.08
4.08
3.00
3.93
4.01
6.09
f1 (ppm)

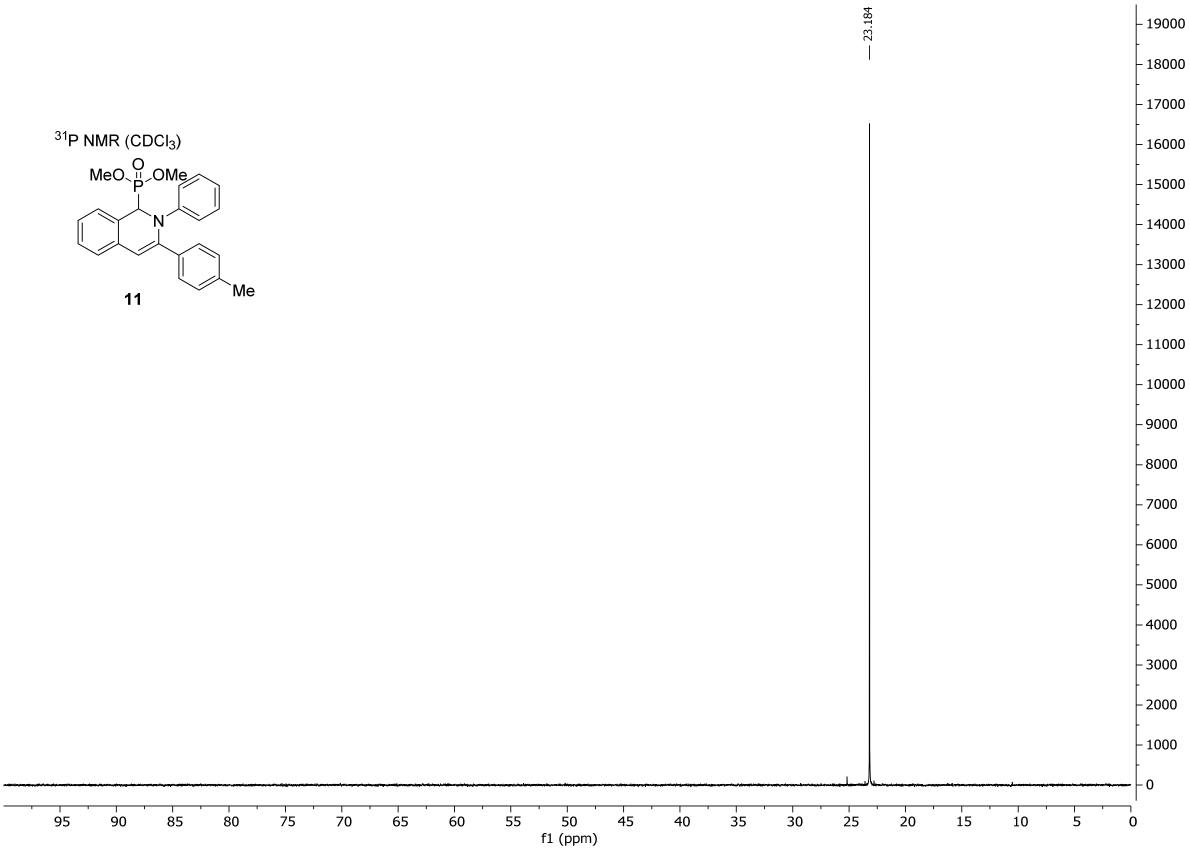
23.184
31P NMR (CDCl3)
MeO
O
P
OMe
N
Me
11
f1 (ppm)

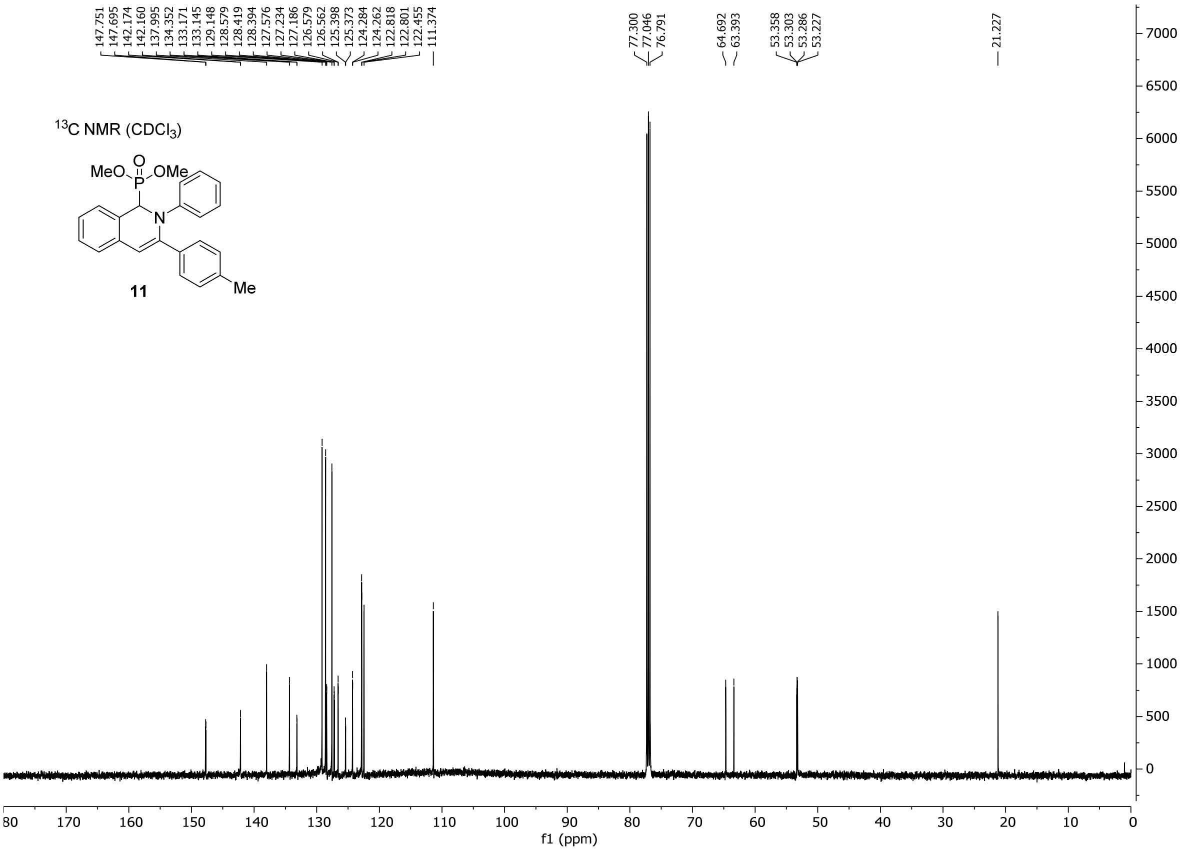
147.751
147.695
142.174
142.160
137.995
134.352
133.171
133.145
129.148
128.579
128.419
128.394
127.576
127.234
127.186
126.579
126.562
125.398
125.373
124.284
124.262
122.818
122.801
122.455
111.374
77.300
77.046
76.791
64.692
63.393
53.358
53.303
53.286
53.227
21.227
13C NMR (CDCl3)
MeO
O
P
OMe
N
Me
11
f1 (ppm)

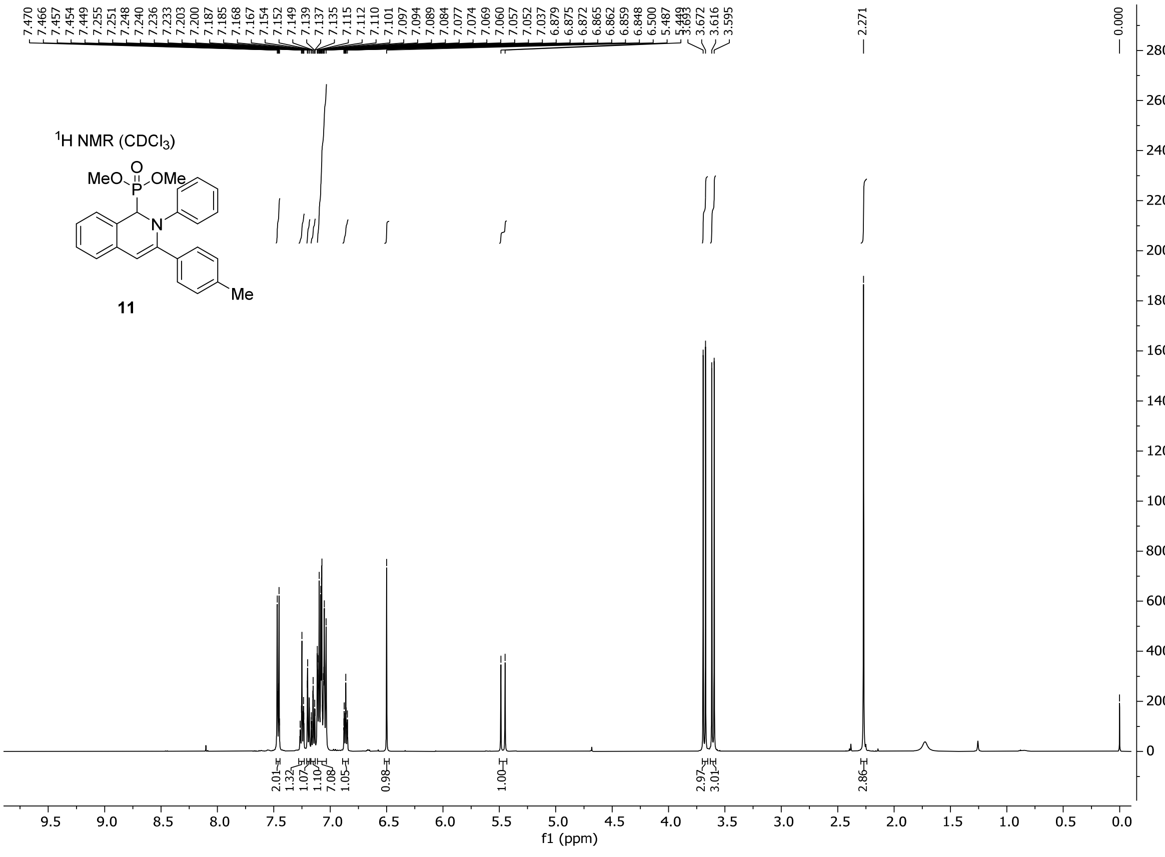

1H NMR (CDCl3)
MeO
OMe
O
P
N
Me
11
7.470 7.466 7.457 7.454 7.449 7.255 7.251 7.248 7.240 7.236 7.233 7.203 7.200 7.187 7.185 7.168 7.167 7.154 7.152 7.149 7.139 7.137 7.135 7.115 7.112 7.110 7.101 7.097 7.094 7.089 7.084 7.077 7.074 7.069 7.060 7.057 7.052 7.037 6.879 6.875 6.872 6.865 6.862 6.859 6.848 6.500 5.487 5.449 3.693 3.672 3.616 3.595
2.271
0.000
2.01 1.32 1.07 1.10 7.08 1.05 0.98 1.00 2.97 3.01 2.86
f1 (ppm)

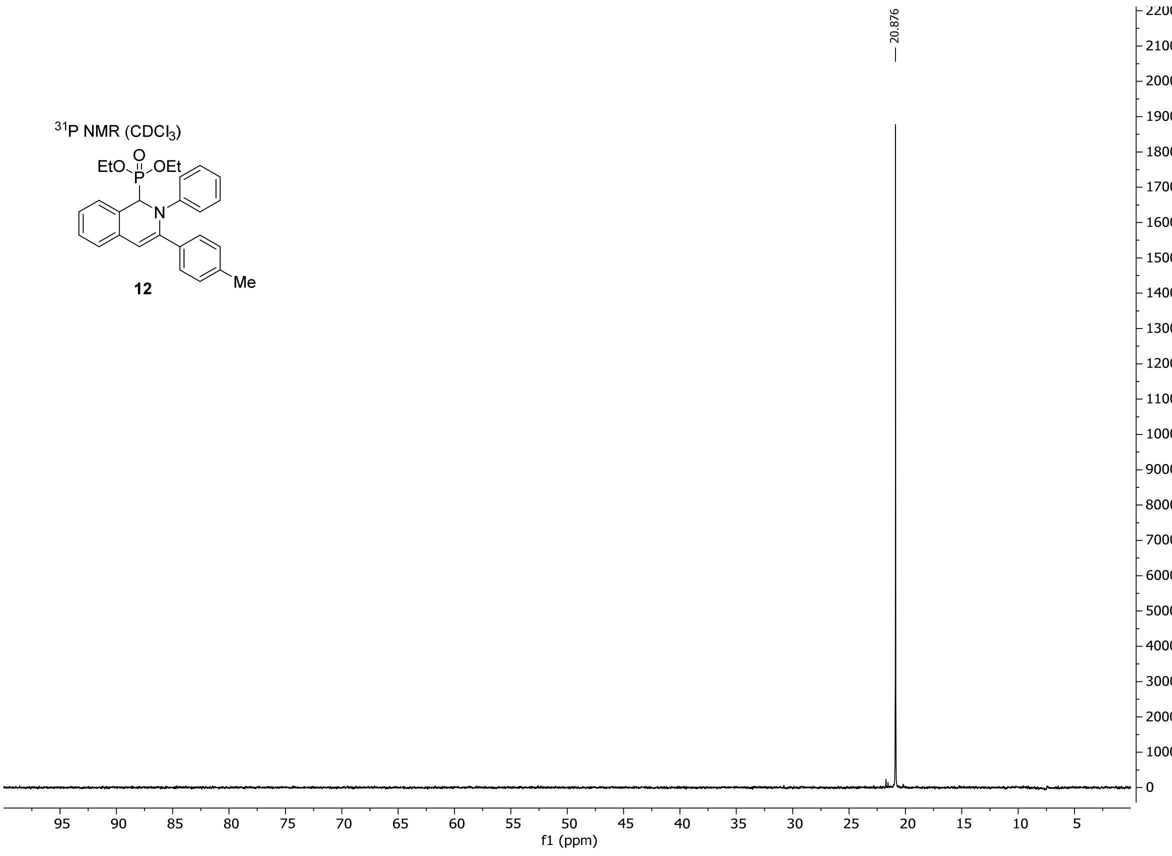

31P NMR (CDCl3)
EtO
OEt
O
P
N
Me
12
20.876
f1 (ppm)

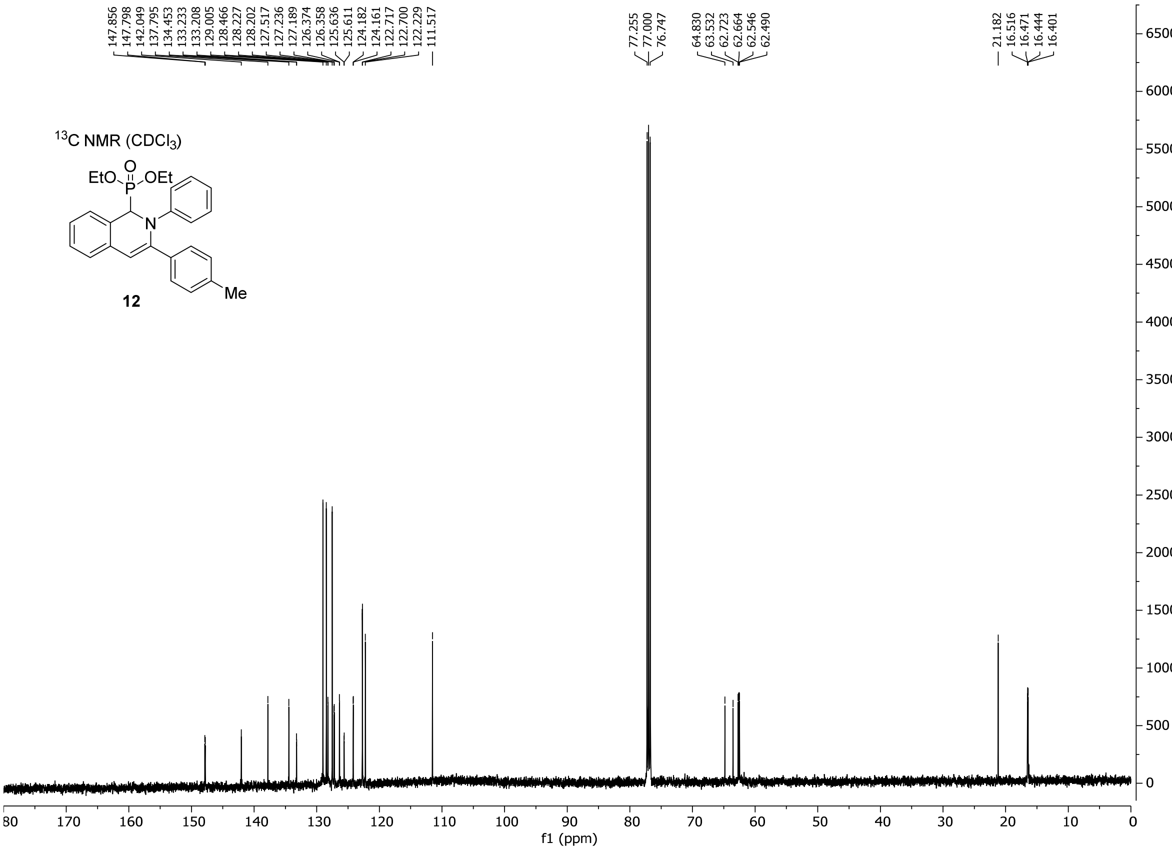
^{13}C NMR (CDCl$_3$)
12
f1 (ppm)

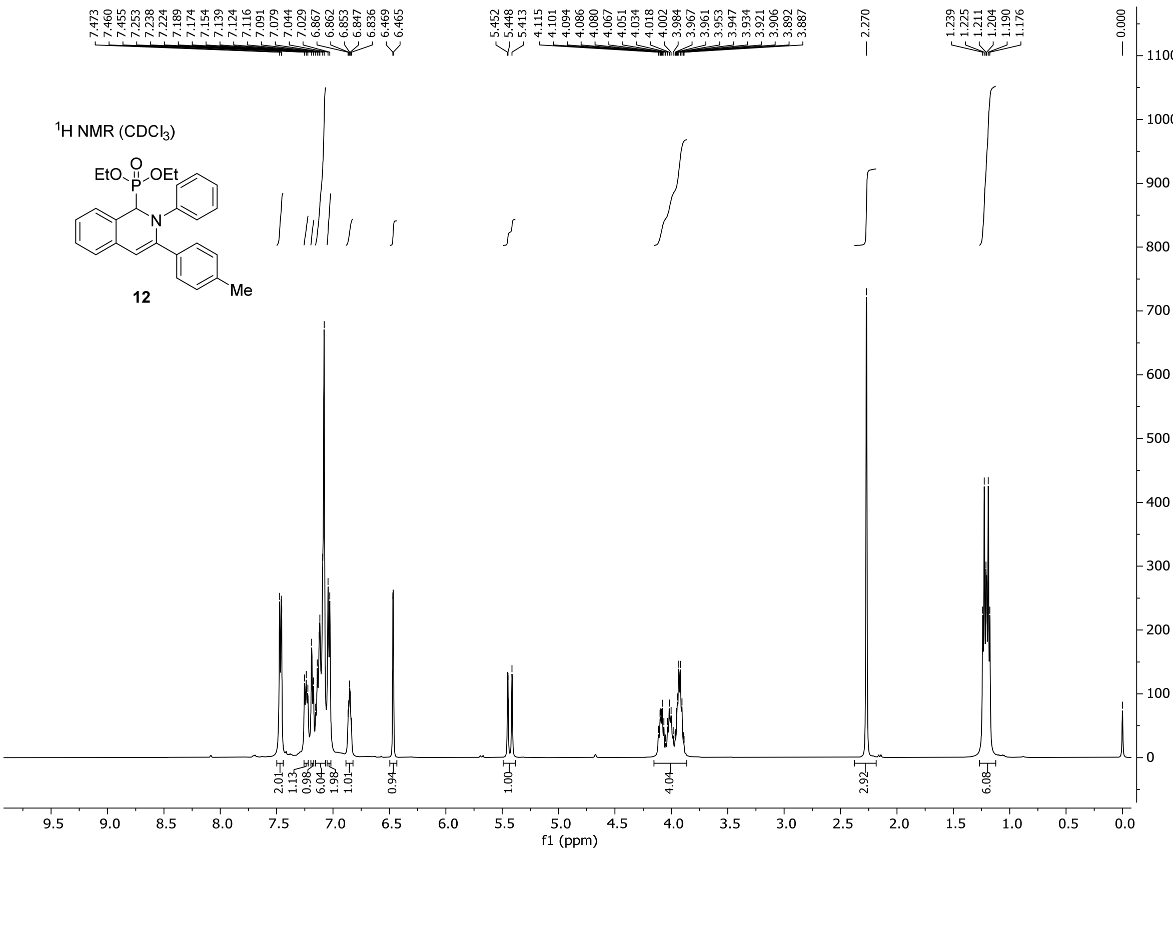
^{1}H NMR (CDCl$_3$)
12
f1 (ppm)

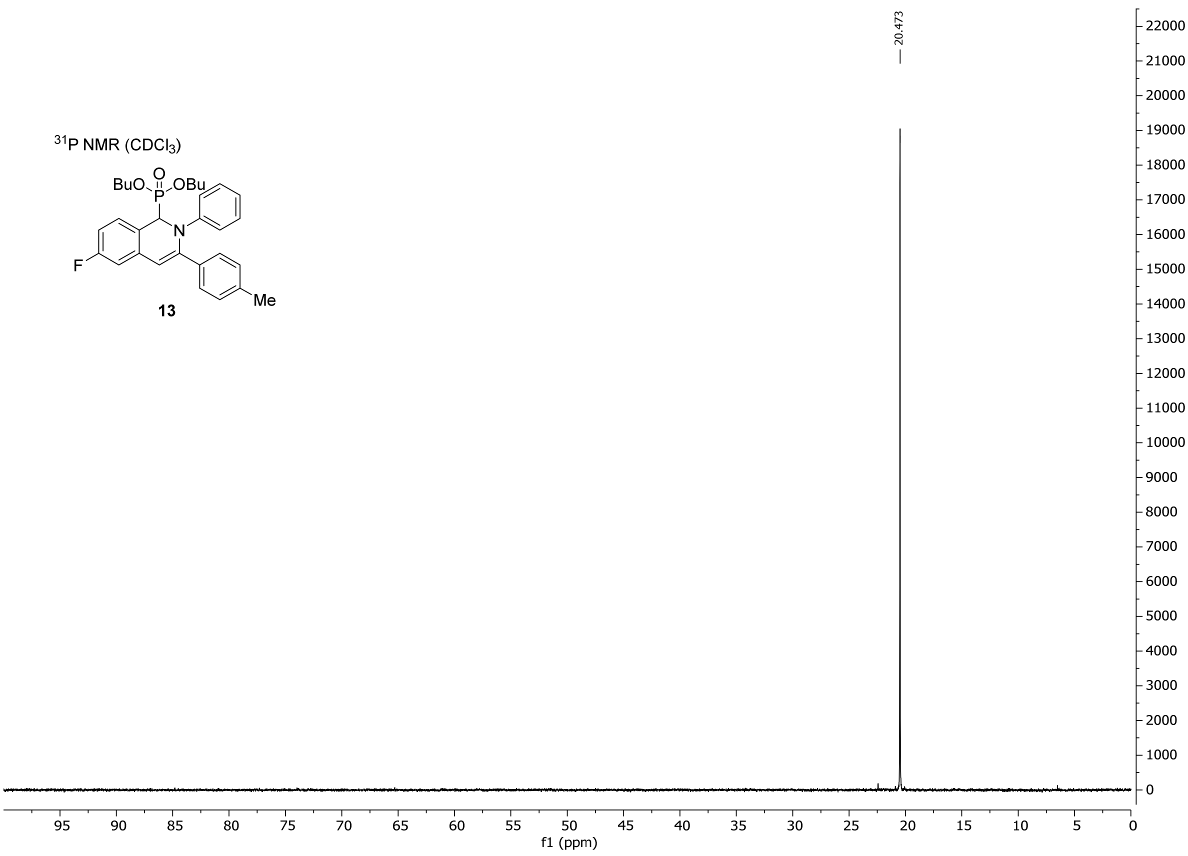

^{31}P NMR ($CDCl_3$)
BuO
OBu
N
F
Me
13
20.473
f1 (ppm)

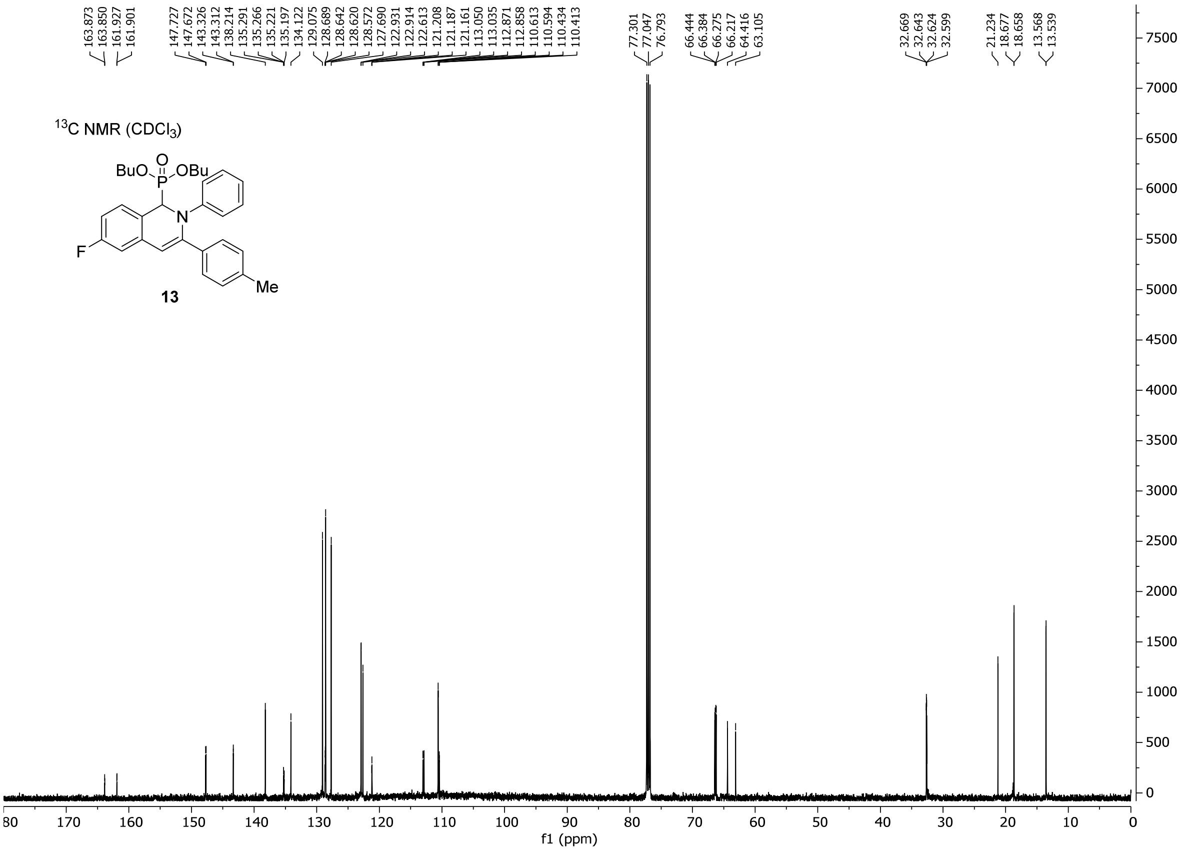

^{13}C NMR ($CDCl_3$)
BuO
OBu
N
F
Me
13
163.873
163.850
161.927
161.901
147.727
147.672
143.326
143.312
138.214
135.291
135.266
135.221
135.197
134.122
129.075
128.689
128.642
128.620
128.572
127.690
122.931
122.914
122.613
121.208
121.187
121.161
113.050
113.035
112.871
112.858
110.613
110.594
110.434
110.413
77.301
77.047
76.793
66.444
66.384
66.275
66.217
64.416
63.105
32.669
32.643
32.624
32.599
21.234
18.677
18.658
13.568
13.539
f1 (ppm)

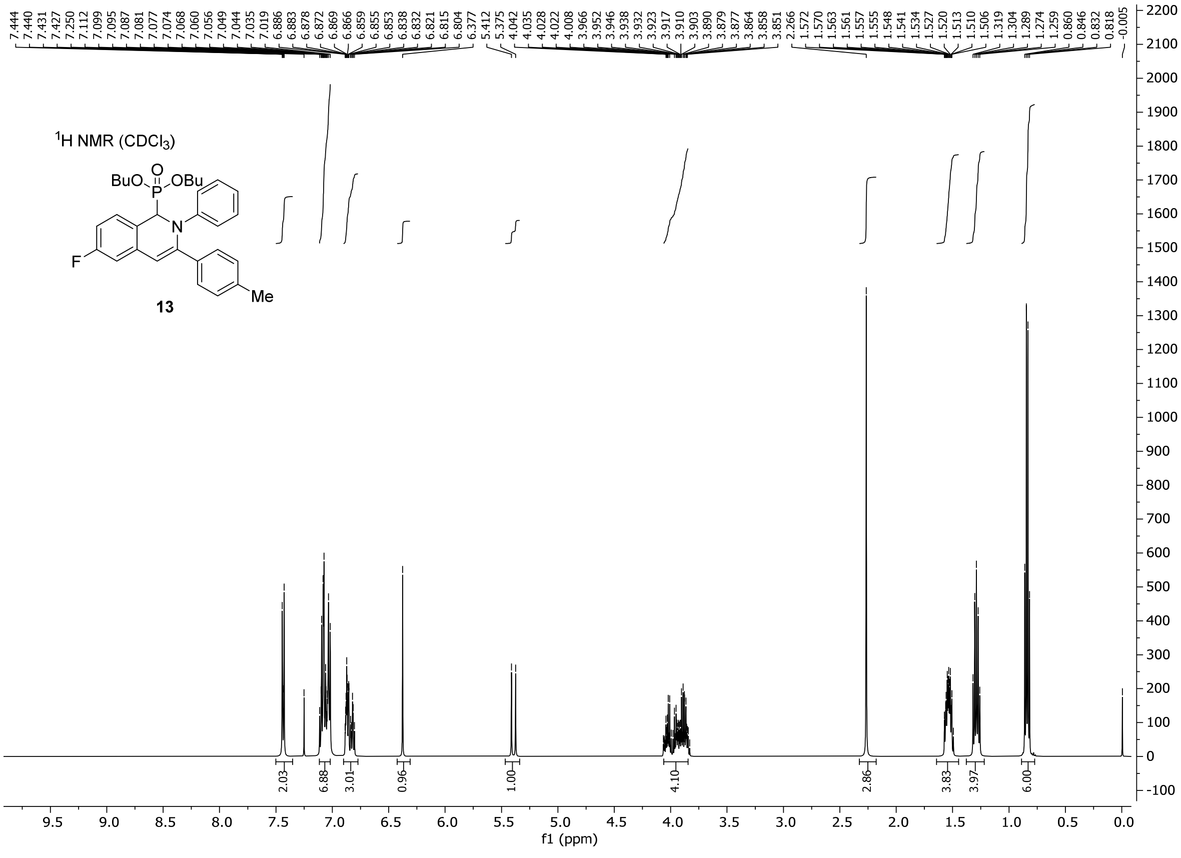
1H NMR (CDCl3)
BuO
O
P
OBu
N
F
Me
13
f1 (ppm)

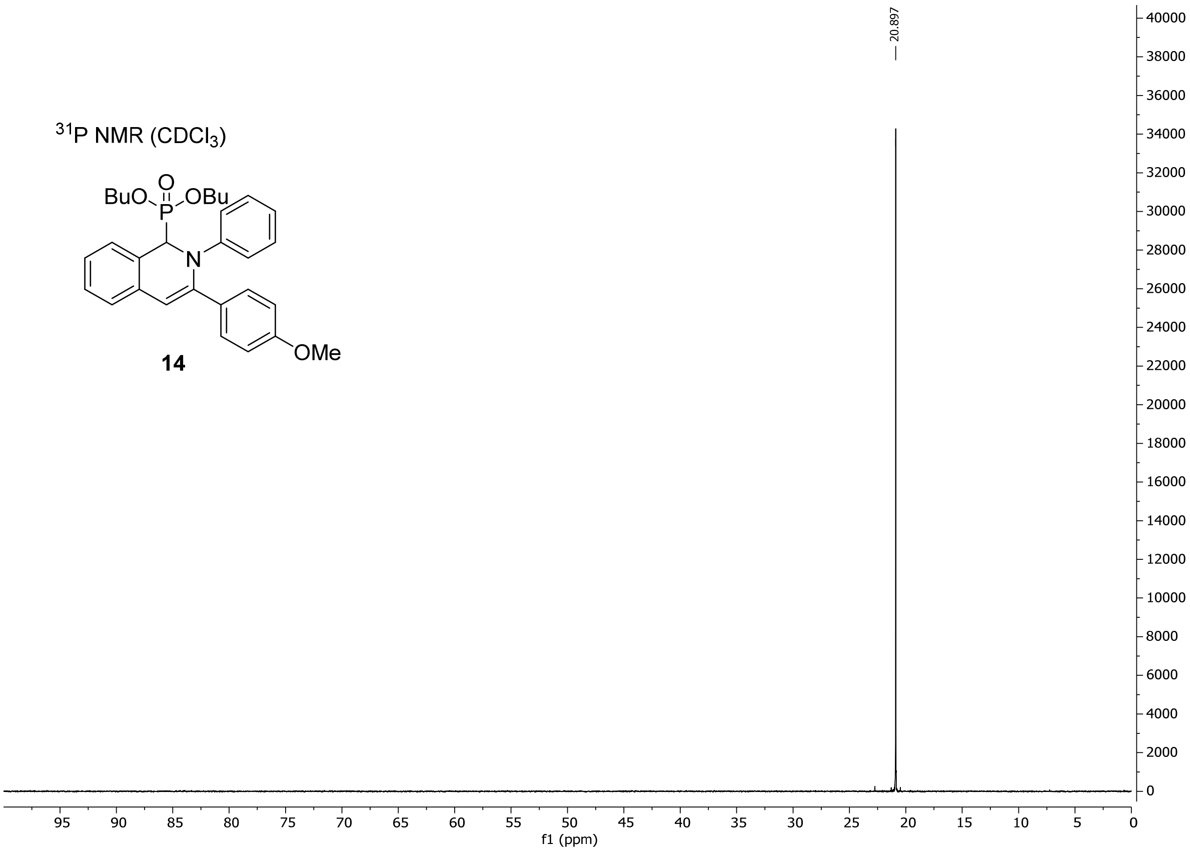
31P NMR (CDCl3)
BuO
O
P
OBu
N
OMe
14
20.897
f1 (ppm)

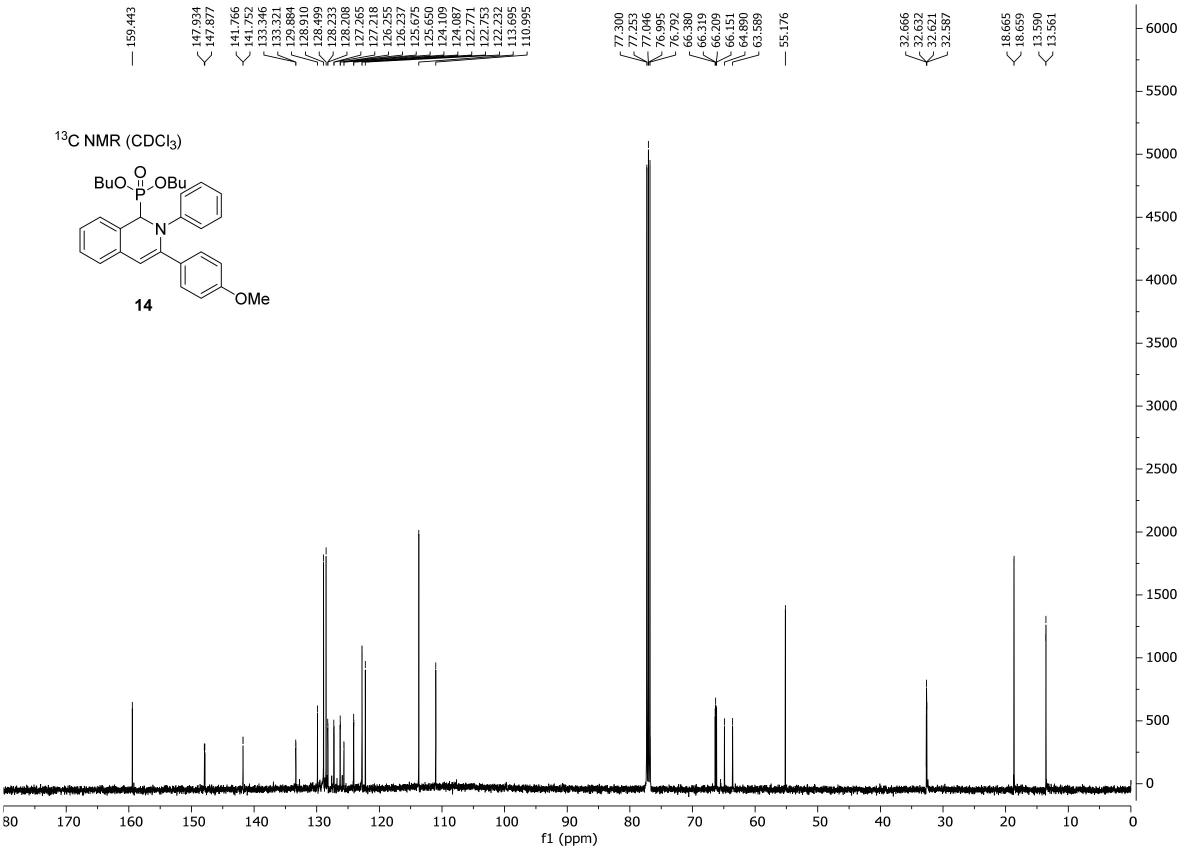
13C NMR (CDCl3)
14
159.443
147.934 147.877
141.766 141.752
133.346 133.321 129.884 128.910 128.499 128.233 128.208 127.265 127.218 126.255 126.237 125.675 125.650 124.109 124.087 122.771 122.753 122.232 113.695 110.995
77.300 77.253 77.046 76.995 76.792 66.380 66.319 66.209 66.151 64.890 63.589
55.176
32.666 32.632 32.621 32.587
18.665 18.659 13.590 13.561
f1 (ppm)

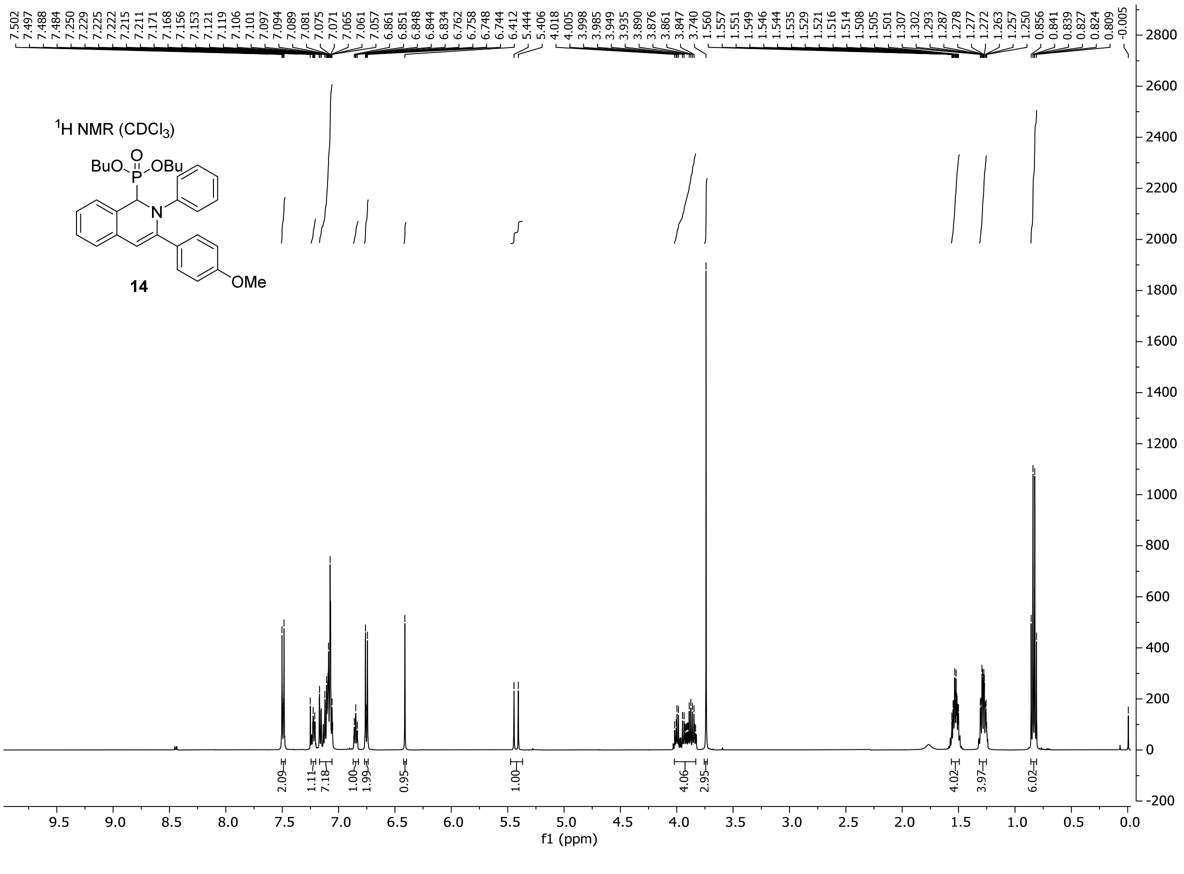
1H NMR (CDCl3)
14
7.502 7.497 7.488 7.484 7.250 7.229 7.225 7.222 7.215 7.211 7.171 7.168 7.156 7.153 7.121 7.119 7.106 7.101 7.097 7.094 7.089 7.081 7.075 7.071 7.065 7.061 7.057 6.861 6.851 6.848 6.844 6.834 6.762 6.758 6.748 6.744 6.412 5.444 5.406 4.018 4.005 3.998 3.985 3.949 3.935 3.890 3.876 3.861 3.847 3.740 1.560 1.557 1.551 1.549 1.546 1.544 1.535 1.529 1.521 1.516 1.514 1.508 1.505 1.501 1.307 1.302 1.293 1.288 1.278 1.277 1.272 1.263 1.257 1.250 0.856 0.841 0.839 0.827 0.824 0.809 -0.005
2.09 1.11 7.18 1.00 1.99 0.95 1.00 4.06 2.95 4.02 3.97 6.02
f1 (ppm)

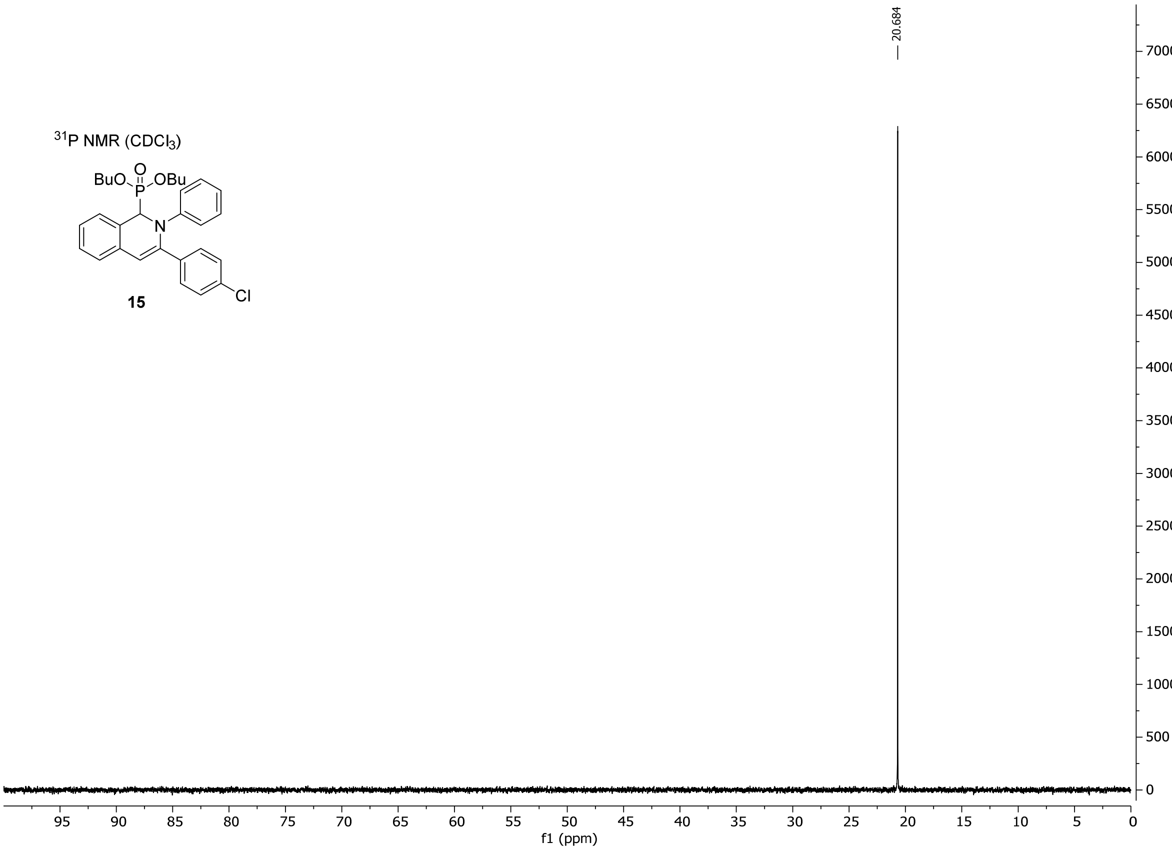
20.684
31P NMR (CDCl3)
BuO
O
P
OBu
N
Cl
15
f1 (ppm)

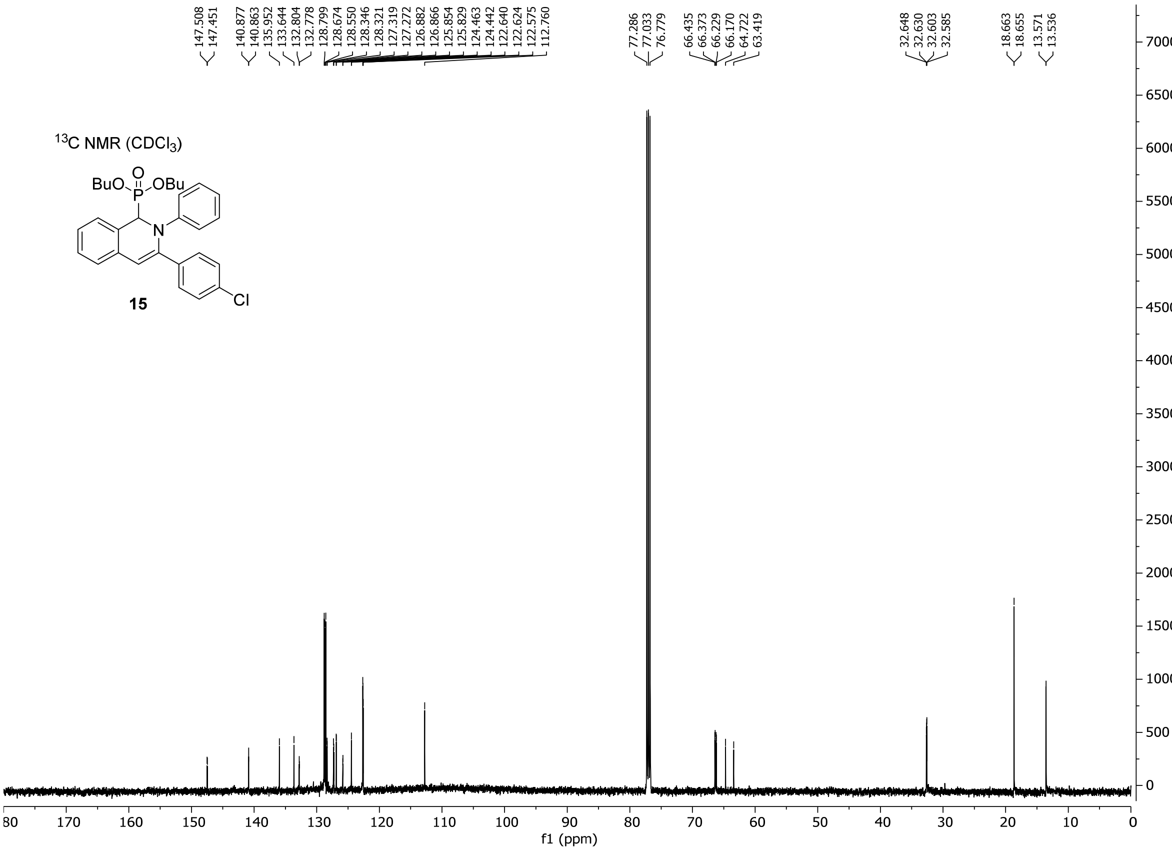
147.508
147.451
140.877
140.863
135.952
133.644
132.804
132.778
128.799
128.674
128.550
128.346
128.321
127.319
127.272
126.882
126.866
125.854
125.829
124.463
124.442
122.640
122.624
122.575
112.760
77.286
77.033
76.779
66.435
66.373
66.229
66.170
64.722
63.419
32.648
32.630
32.603
32.585
18.663
18.655
13.571
13.536
13C NMR (CDCl3)
BuO
O
P
OBu
N
Cl
15
f1 (ppm)

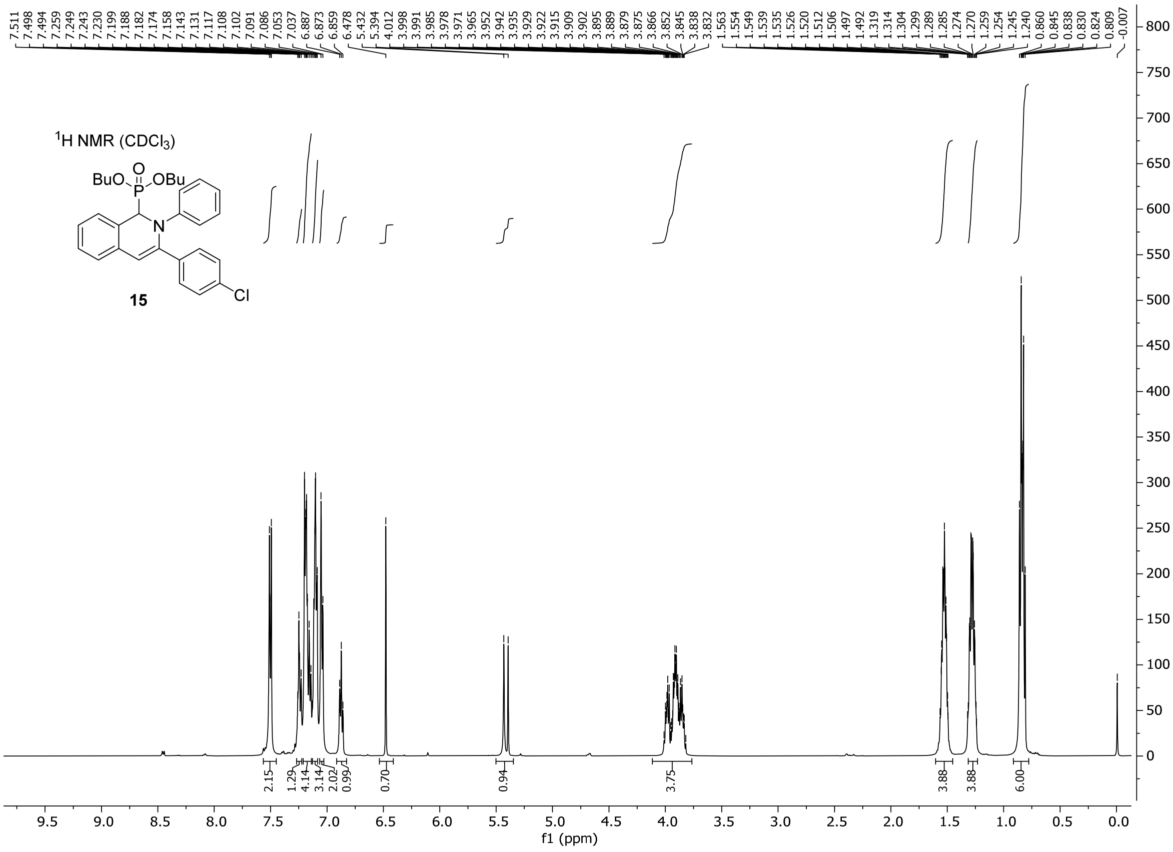

1H NMR (CDCl3)
BuO
O
P
OBu
N
Cl
f1 (ppm)

15

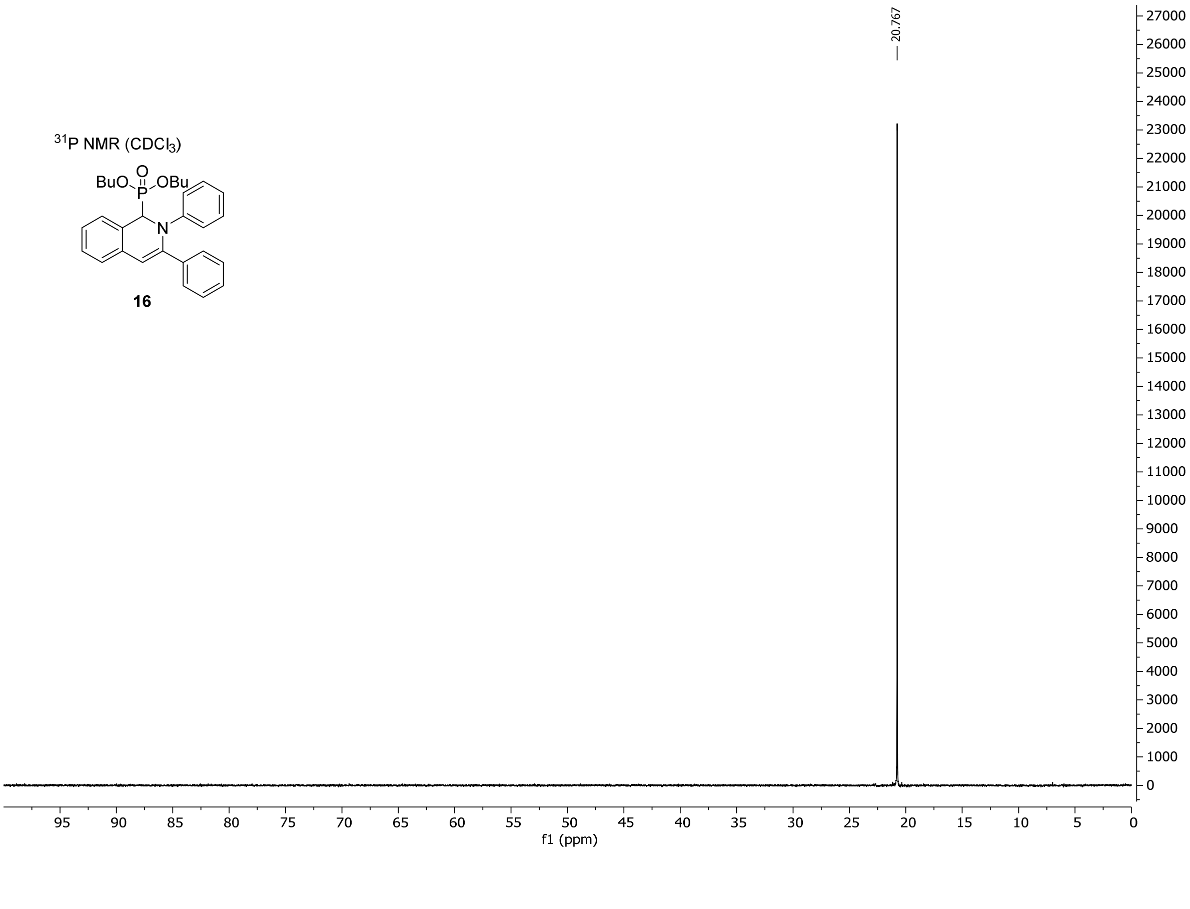

31P NMR (CDCl3)
BuO
O
P
OBu
N
20.767
f1 (ppm)

16

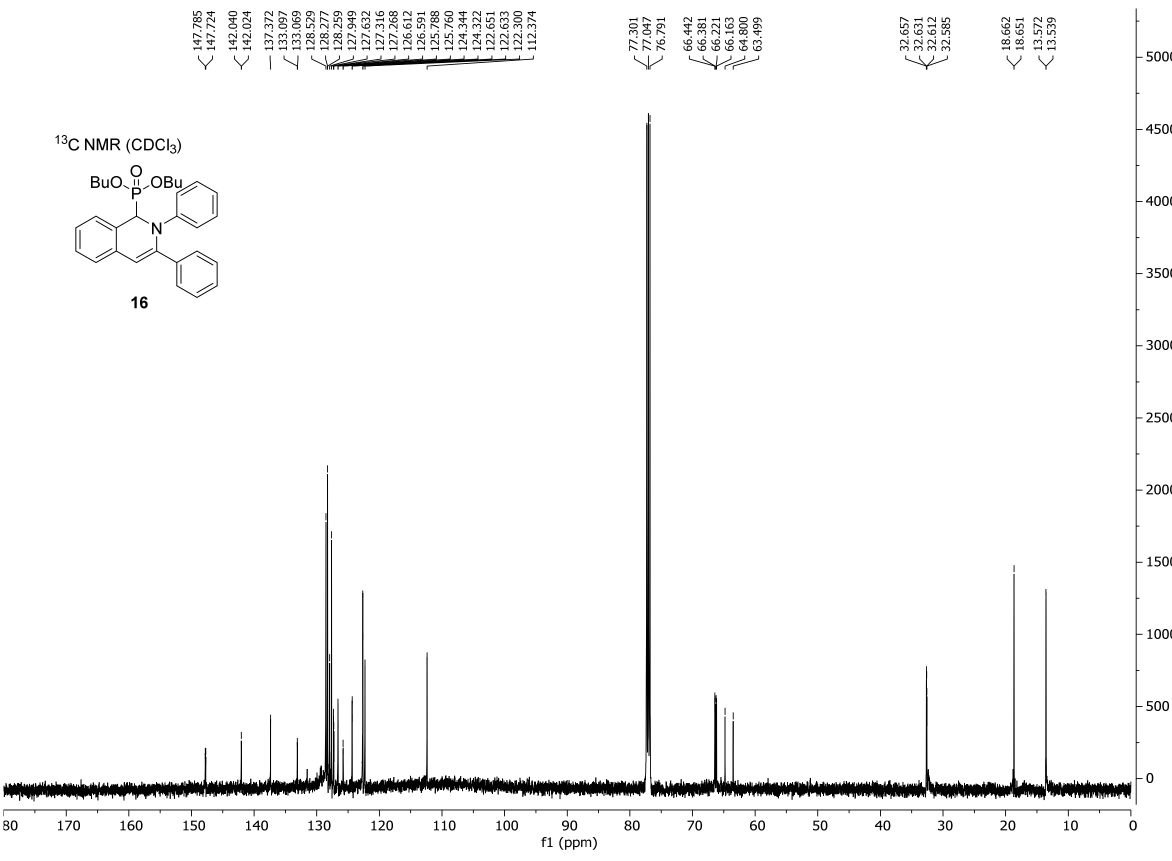
147.785
147.724
142.040
142.024
137.372
133.097
133.069
128.529
128.277
128.259
127.949
127.632
127.316
127.268
126.612
126.591
125.760
125.788
124.344
124.322
122.651
122.633
122.300
112.374
77.301
77.047
76.791
66.442
66.381
66.221
66.163
64.800
63.499
32.657
32.631
32.612
32.585
18.662
18.651
13.572
13.539
13C NMR (CDCl3)
BuO
O
P
OBu
N
16
f1 (ppm)

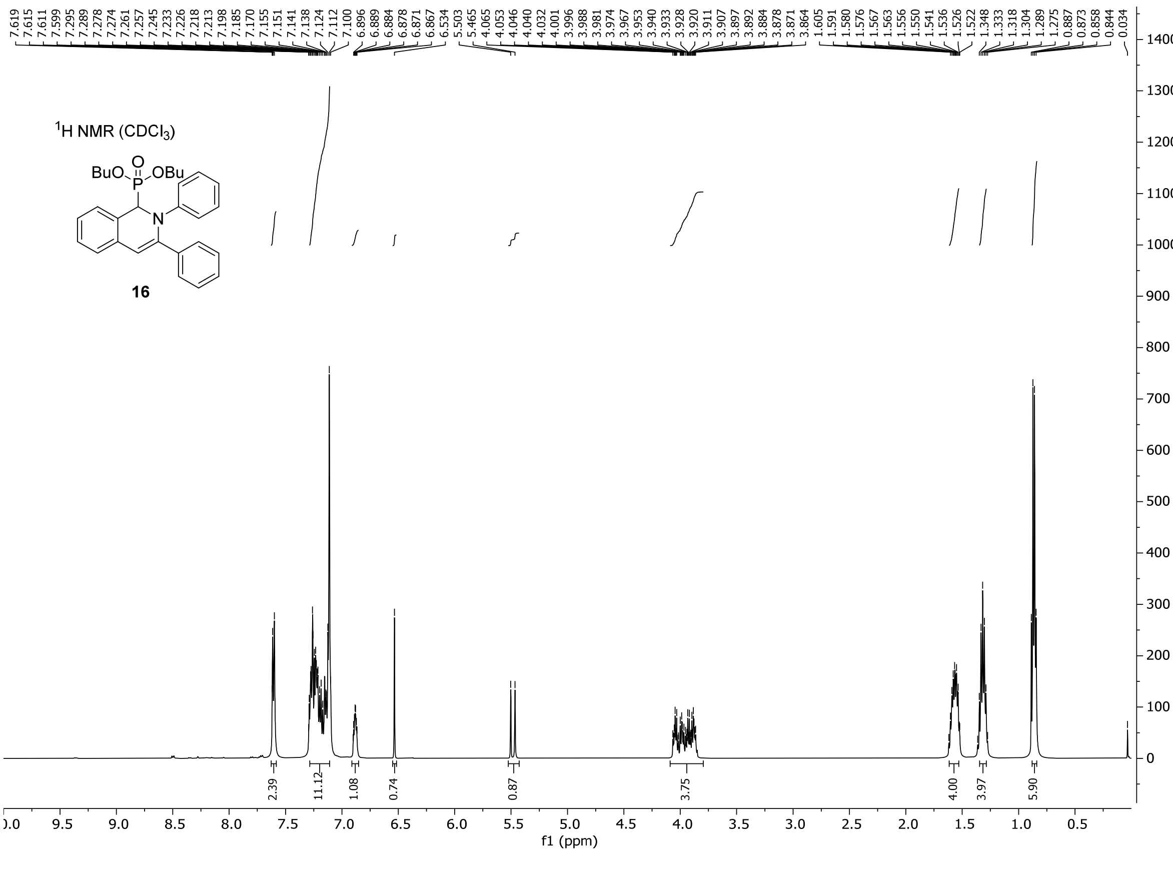
7.619
7.615
7.611
7.599
7.295
7.289
7.278
7.274
7.261
7.257
7.245
7.233
7.226
7.218
7.213
7.198
7.185
7.170
7.155
7.151
7.141
7.138
7.124
7.112
7.100
6.896
6.889
6.884
6.878
6.871
6.867
6.534
5.503
5.465
4.065
4.053
4.046
4.040
4.032
4.001
3.996
3.988
3.981
3.974
3.967
3.953
3.940
3.933
3.928
3.920
3.911
3.907
3.897
3.892
3.884
3.878
3.871
3.864
1.605
1.591
1.580
1.576
1.567
1.563
1.556
1.550
1.541
1.536
1.526
1.522
1.348
1.333
1.318
1.304
1.289
1.275
0.887
0.873
0.858
0.844
0.034
1H NMR (CDCl3)
BuO
O
P
OBu
N
16
2.39
11.12
1.08
0.74
0.87
3.75
4.00
3.97
5.90
f1 (ppm)
